# The application of transcranial magnetic stimulation in treatment-resistant depression: insights from omics

**DOI:** 10.3389/fpsyt.2026.1938107

**Published:** 2026-10-20

**Authors:** Yuan Yang, Xiaolina Zhang, Yushan Duan, Jie Wu, Mengyuan Zhang, Ruize Niu, Tianhao Bao

**Affiliations:** 1 Psychiatric Hospital of Yunnan Province, The Affiliated Mental Health Center of Kunming Medical University, Kunming, China; 2 Department of Critical Care Medicine, The Second Affiliated Hospital, Kunming Medical University, Kunming, China

**Keywords:** biomarkers, multi-omics, neuromodulation, precision psychiatry, transcranial magnetic stimulation, treatment-resistant depression

## Abstract

Treatment-resistant depression (TRD) is a refractory form of major depressive disorder (MDD) for which conventional antidepressant strategies are often insufficient. Transcranial magnetic stimulation (TMS) has demonstrated clinical efficacy in TRD, but its biological mechanisms remain incompletely understood. This narrative review synthesizes current evidence across transcriptomic, epigenomic, proteomic, metabolomic, and connectomic levels and is complemented by exploratory secondary analyses of publicly available omics datasets. Transcriptomic and spatial transcriptomic studies suggest that TMS modulates cell type- and region-specific transcriptional programs related to synaptic plasticity and neuronal development. Epigenetic evidence suggests potential roles for DNA methylation and lactylation-related regulation, whereas proteomic and metabolomic studies indicate changes in synaptic proteins, neurotransmitter systems, lipid metabolism, and peripheral metabolites. Connectomic studies further show that TMS can alter functional and structural connectivity involving prefrontal and large-scale brain networks associated with treatment response. Together, current evidence indicates biological changes across multiple levels, although direct causal links between molecular changes and circuit remodeling remain largely unproven. Future longitudinal multi-omics studies in patients with TRD may help identify biomarkers, clarify mechanisms, and improve individualized TMS treatment.

## 1 Introduction

Major depressive disorder (MDD) is a common psychiatric disorder and one of the leading causes of disability worldwide. Although conventional antidepressants, manualized psychotherapy, and certain forms of neurostimulation have demonstrated efficacy, most patients with MDD show an inadequate response to first-line treatment. A substantial proportion of these patients fail to respond to multiple antidepressant interventions, resulting in what is termed treatment-resistant depression (TRD) (1). At present, no universally precise definition of TRD exists. The definitions adopted by the U.S. Food and Drug Administration (FDA) and the European Medicines Agency (EMA) characterize TRD as nonresponse to at least two adequate antidepressant regimens despite sufficient dose, duration, and adherence (2).

Transcranial magnetic stimulation (TMS) is a noninvasive focal brain stimulation technique based on electromagnetic induction, in which fluctuating magnetic fields generate weak intracranial currents. In 1985, TMS was introduced into neuroscience as a noninvasive technique capable of eliciting motor responses through direct magnetic stimulation of the motor cortex, thereby allowing assessment of neural connectivity and excitability (3). Over the past four decades, TMS has been widely used in the treatment of neurological and psychiatric disorders, including autism spectrum disorder, schizophrenia, depression, treatment-resistant psychosis, traumatic brain injury, Alzheimer’s disease, Parkinson’s disease, and diabetes-related neurological dysfunction (4). TMS modalities currently used or investigated for depression include conventional repetitive transcranial magnetic stimulation (rTMS) and intermittent theta-burst stimulation (iTBS), which are established treatment protocols; deep transcranial magnetic stimulation (dTMS), which is used clinically for depression; continuous theta-burst stimulation (cTBS), which remains investigational for depression; and accelerated transcranial magnetic stimulation (aTMS) protocols, such as Stanford Neuromodulation Therapy (SNT), a specific accelerated iTBS protocol that delivers multiple sessions per day to shorten the treatment course (5, 6). The mechanisms and clinical efficacy of these modalities differ. In 1993, TMS was first shown to exert a modest therapeutic effect in patients with treatment-resistant MDD (7). Since then, a large body of clinical evidence has demonstrated favorable efficacy of TMS in TRD (8–13). Compared with pharmacotherapy, TMS is widely recognized as a cost-effective treatment option for TRD (14). Studies have shown that TMS can improve rating-scale scores (15, 16), sleep quality (17, 18), cognitive function (19–22), inflammatory status (23–25), and frontal cerebral blood volume (26) in patients with TRD. TMS may influence brain function in depression through multiple mechanisms, including modulation of neurotransmitter and receptor synthesis and release (27–33), neurotrophic factor production (34–38), synapse-related molecules (29), neural regeneration (39–41), inflammatory mediator synthesis and release (24, 25, 42), programmed cell death (40, 43, 44), oxidative stress (45–47), tissue metabolism (27), epigenetic regulation (48), neural conduction (35, 49), and functional brain networks (50). However, most previous studies have relied on low-throughput approaches to explore the mechanisms of TMS in depression, and a comprehensive omics-based understanding remains lacking.

Omics comprises a collection of technologies and disciplines for the systematic study of diverse biomolecules and their interactions within biological systems. These approaches include transcriptomics, translatomics, proteomics, metabolomics, epigenomics, and microbiomics. Connectomics refers to the mapping and analysis of structural and functional brain connectivity; in psychiatric research, connectivity networks are reconstructed or characterized using measurements obtained with functional magnetic resonance imaging (fMRI), diffusion tensor imaging (DTI), electroencephalography (EEG), and positron emission tomography (PET). Multiple omics technologies have been used to investigate the pathophysiology of MDD and TRD and the potential therapeutic mechanisms of TMS. Nevertheless, omics studies specifically addressing TMS in TRD remain limited, and much of the available evidence is derived from broader MDD populations, preclinical studies, or imaging-based connectivity analyses. To identify relevant evidence, we searched PubMed, Web of Science, and the Cochrane Library using TMS-, depression-, and omics-related terms; the search strategy, eligibility criteria, and final search date are described in Section 2.1. The following sections summarize the available evidence and distinguish direct clinical findings from broader MDD, preclinical, and hypothesis-generating evidence where appropriate.

## 2 Materials and methods

### 2.1 Literature search and study selection

Although this study is a narrative review, a structured approach was adopted to enhance the transparency and reproducibility of the literature selection process.

Relevant studies were retrieved from PubMed, Web of Science, and the Cochrane Library, with the final search conducted on March 10, 2025. The search strategy combined keywords related to transcranial magnetic stimulation and depression, including “transcranial magnetic stimulation”, “TMS”, “repetitive transcranial magnetic stimulation”, “rTMS”, “treatment-resistant depression”, and “depression”, together with omics-related terms such as “transcriptomics”, “epigenomics”, “proteomics”, “metabolomics”, and “connectomics”.

Studies were included if they: (1) involved patients with MDD/TRD or relevant animal models; (2) investigated TMS interventions or omics-related mechanisms; and (3) were published in English. Studies were excluded if full texts were unavailable, data could not be extracted, or the publication type was non-peer-reviewed.

Literature screening and data extraction were conducted by two independent reviewers, and discrepancies were resolved through discussion. Study selection was primarily based on relevance to the topic, methodological quality, and contribution to the understanding of TMS mechanisms.

Given the heterogeneity of included studies (including experimental and omics-based research), a formal risk-of-bias assessment and PRISMA-based systematic review protocol were not applied. However, efforts were made to ensure a comprehensive and balanced synthesis of the current evidence.

### 2.2 Secondary analyses of publicly available omics data

To illustrate how existing multi-omics data can be integrated to generate mechanistic hypotheses, we performed exploratory secondary analyses of publicly available datasets. Human prefrontal cortex (PFC) single-nucleus RNA-seq data from patients with MDD and controls were obtained from GSE144136 and GSE213982, and cortical RNA-seq data from TMS-stimulated rodents were obtained from GSE230149. Data were preprocessed and normalized according to defined pipelines; cell types were annotated using established marker genes; differentially expressed genes were identified using prespecified statistical thresholds; and directional overlap between TMS-induced and MDD-related transcriptional changes was examined across cell types. Published spatial transcriptomic and proteomic findings were integrated with the MDD and TMS transcriptomic results using direction-aware cross-dataset comparisons, whereas cell type-specific metabolic alterations were inferred from single-cell transcriptomic data using scFEA. Functional enrichment analysis was performed on candidate directional-overlap gene set**s**. All secondary analyses were exploratory and hypothesis-generating. Full computational details, including software versions, parameters, statistical thresholds, cross-species ortholog mapping, proteomic integration, and metabolic inference, are provided in the **Supplementary Methods**.

## 3 Results

### 3.1 Applications of bulk RNA sequencing and single-cell transcriptomics

Evidence sources: (i) clinical transcriptomic studies in MDD; (ii) preclinical TMS studies in rodents; and (iii) exploratory integration of publicly available datasets. The latter analyses were performed for hypothesis generation and should not be interpreted as confirmatory donor-level evidence. Single-cell RNA sequencing (scRNA-seq) can resolve cell type-specific transcriptomic alterations in complex neuropsychiatric disorders (51–53). Studies have shown that transcriptomic changes occur in 60% of cells in the PFC of patients with MDD (54). Distinct subpopulations of excitatory neurons, inhibitory neurons, and oligodendrocyte precursor cells (OPCs), among other cell types, are affected to varying degrees (54). The most severe dysregulation occurs in deep-layer excitatory neurons and immature OPCs, which together account for nearly half (47%) of all gene expression changes. Notably, 5-Hz rTMS can influence oligodendrocytes by increasing the number of Olig2-positive cells in the hilus of the dentate gyrus and in the PFC, thereby reversing depression-like behaviors in mice exposed to chronic mild stress (55). This finding may partly explain the therapeutic mechanism of TMS. These results underscore the importance of anatomically and cell type-specific contributions to disease and create opportunities to identify new research avenues and therapeutic targets. In addition, these transcriptomic changes show marked sex differences (56). In females, microglia and parvalbumin-positive (PVALB) interneurons are most strongly affected, whereas in males the greatest changes are observed in deep-layer excitatory neurons, astrocytes, and OPCs.

RNA sequencing (RNA-seq) results indicate that the overall effect of iTBS and cTBS on the sensorimotor cortical transcriptome of healthy mice 3 hours after stimulation is very modest (293 differentially expressed genes, of which only 12 are annotated protein-coding genes; false discovery rate [FDR] < 0.05 and |log2 fold change [log2FC]| > 1) (57). Several explanations are possible: (1) the duration and number of stimulation pulses (600 pulses) may have been insufficient, yielding cumulative effects too small to detect; (2) TMS may induce transcriptional changes only at finer spatial or cellular resolution, whereas bulk sequencing is insufficiently sensitive to capture those signals; or (3) TMS may selectively regulate diseased rather than healthy brains, such that healthy brain tissue responds weakly or sluggishly, whereas neural cells under disease conditions may be more susceptible to electrical or magnetic stimulation. Another study in healthy rats, using a higher pulse number (3,000 pulses), found a more pronounced overall cortical effect (321 differentially expressed genes, including 149 annotated protein-coding genes; FDR < 0.05 and |log2FC| > 1) (58). Although this comparison suggests that stimulation parameters may influence the magnitude of transcriptional changes—an interpretation supported by a study showing that repeated rTMS sessions (81 differentially expressed genes) produced more gene expression changes than a single session (25 differentially expressed genes) (59)—differences across studies may also reflect species, brain region, sampling time, sequencing approach, sample size, and statistical thresholds, and the available data do not by themselves establish a dose-dependent transcriptional effect.

To explore potential convergent transcriptional signals related to TMS and MDD, we conducted an exploratory cross-dataset integration of scRNA-seq data from patients with MDD (GSE144136 and GSE213982) and cortical RNA-seq data from TMS-stimulated rats (GSE230149). We identified directional overlap between TMS-responsive cortical genes and cell-level transcriptional signals observed across multiple cortical cell types in MDD (**Figures 1A, B**). Because the MDD differential-expression analysis was performed at the cell/nucleus level without explicit donor-level modeling, these overlaps should be interpreted as exploratory and hypothesis-generating rather than as confirmatory subject-level evidence. Among the exploratory directional-overlap signals, upregulated genes were more frequently observed, and excitatory and inhibitory neurons showed relatively large numbers of overlapping genes (**Figures 1A–C**). DDX17 was upregulated in six cell types (**Figure 1C**). DDX17 is an RNA helicase involved in key processes of early neuronal differentiation. Allelic variation in DDX17 has been associated with neurodevelopmental abnormalities, predominantly intellectual disability, speech and language delay, and motor delay (60). Knockdown of Ddx17 delays neuronal migration in mice and reduces cortical axonal complexity, and deletion of Ddx17 in primary mouse cortical neurons *in vitro* decreases axonal growth (60). WSB1 was upregulated in five cell types (**Figure 1C**). A common genetic form of Parkinson’s disease (PD) is caused by leucine-rich repeat kinase 2 (LRRK2) mutations, and WSB1 is an LRRK2-interacting protein. WSB1 promotes K27- and K29-linked ubiquitination of LRRK2, leading to its aggregation and conferring neuroprotection in primary neurons. In neuronal and Drosophila models, deletion of endogenous WSB1 exacerbates mutant LRRK2 neurotoxicity, indicating that endogenous WSB1 modulates LRRK2-mediated cytotoxicity. WSB1 is also present in Lewy bodies of postmortem human PD tissue, suggesting a role in mutant LRRK2 pathogenesis and possibly in sporadic PD-related Lewy body pathology. Previous studies have shown that rTMS significantly improves motor dysfunction in patients with PD (61, 62). DCLK1 and COL16A1 were upregulated in four cell types (**Figure 1C**). DCLK1 encodes a member of both the protein kinase superfamily and the doublecortin family and participates in multiple cellular processes, including neuronal migration, axonal transport, axon guidance, neuronal apoptosis, and neurogenesis (63–67). DCLK1 can be upregulated by brain-derived neurotrophic factor (BDNF), is associated with memory and general cognitive ability (68), and BDNF itself is an important therapeutic target of rTMS (69–71). The directional overlap of these genes between the exploratory MDD cell-level analysis and TMS-induced transcriptional changes highlights DDX17, WSB1, and DCLK1 as candidate genes for further investigation. However, because donor-level dependence was not modeled in the MDD analysis and the TMS transcriptomic data were obtained from healthy animals, these cross-dataset overlaps should not be interpreted as evidence that these genes mediate clinical TMS effects. Functional enrichment analysis of the exploratory directional-overlap gene sets in excitatory and inhibitory neurons identified biological processes related to synaptic integration, regulation of postsynaptic membrane potential, synaptic structure and activity, and vascular transport (**Figures 1D, E**).

**Figure 1:**
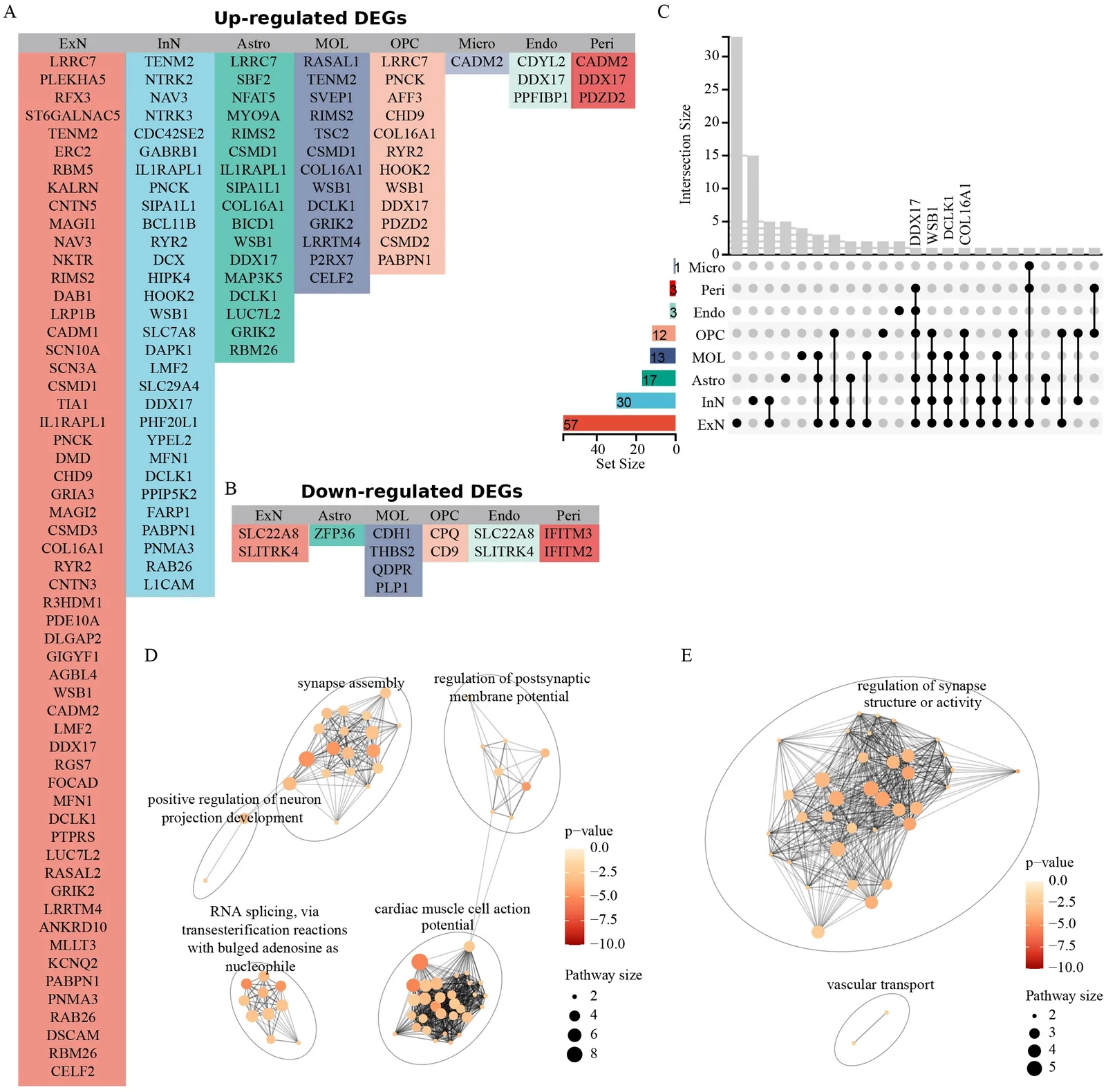
Exploratory integrative transcriptomic analysis identifying directional-overlap genes across cortical cell types. **(A)** Genes up-regulated by TMS that overlap genes down-regulated in the PFC in MDD, shown by cell type; excitatory neurons showed the largest number of overlapping genes. **(B)** Genes down-regulated by TMS that overlap genes up-regulated in MDD, by cell type. **(C)** UpSet plot showing the distribution of candidate directional-overlap genes across cell types; DDX17, WSB1 and DCLK1 were shared by the largest number of cell types. **(D)** Functional enrichment of candidate directional-overlap genes in excitatory neurons. **(E)** Functional enrichment of candidate directional-overlap genes in inhibitory neurons. Genes shared across ≥2 cell types are highlighted in red **(A, B)**. Panels **(D, E)** highlight processes related to synaptic integration, regulation of postsynaptic membrane potential, control of synaptic structure and activity, and vascular transport.

### 3.2 Application of spatial transcriptomics

Evidence sources: preclinical TMS studies in rodents and exploratory integration of publicly available datasets (hypothesis-generating). Because bulk RNA-seq results show that iTBS and cTBS exert only modest overall effects on the transcriptome of the sensorimotor cortex, rTMS may instead induce transcriptional changes at finer resolution, that is, with spatial and substructural specificity. Spatial transcriptomics sequencing (ST-seq) can therefore help resolve TMS-induced transcriptional alterations at higher resolution and characterize changes in cortical and subcortical structures. Studies indicate that the effects of rTMS are heterogeneous across the brain and differ among cortical regions and across cortical layers (57). Overall, cTBS induces a greater number of differentially expressed genes than iTBS. Although the two protocols share some overlapping genes and biological processes, each also produces distinct transcriptomic signatures. By integrating published spatial transcriptomic findings with the exploratory cell-level MDD gene sets, we identified genes showing opposite directional patterns across different cell types and cortical regions (**Figure 2**). These exploratory correspondences highlight potentially relevant spatial and cell type-specific transcriptional patterns, but they do not establish molecular reversal by TMS and require confirmation using donor-aware analyses and independent experimental validation.

**Figure 2:**
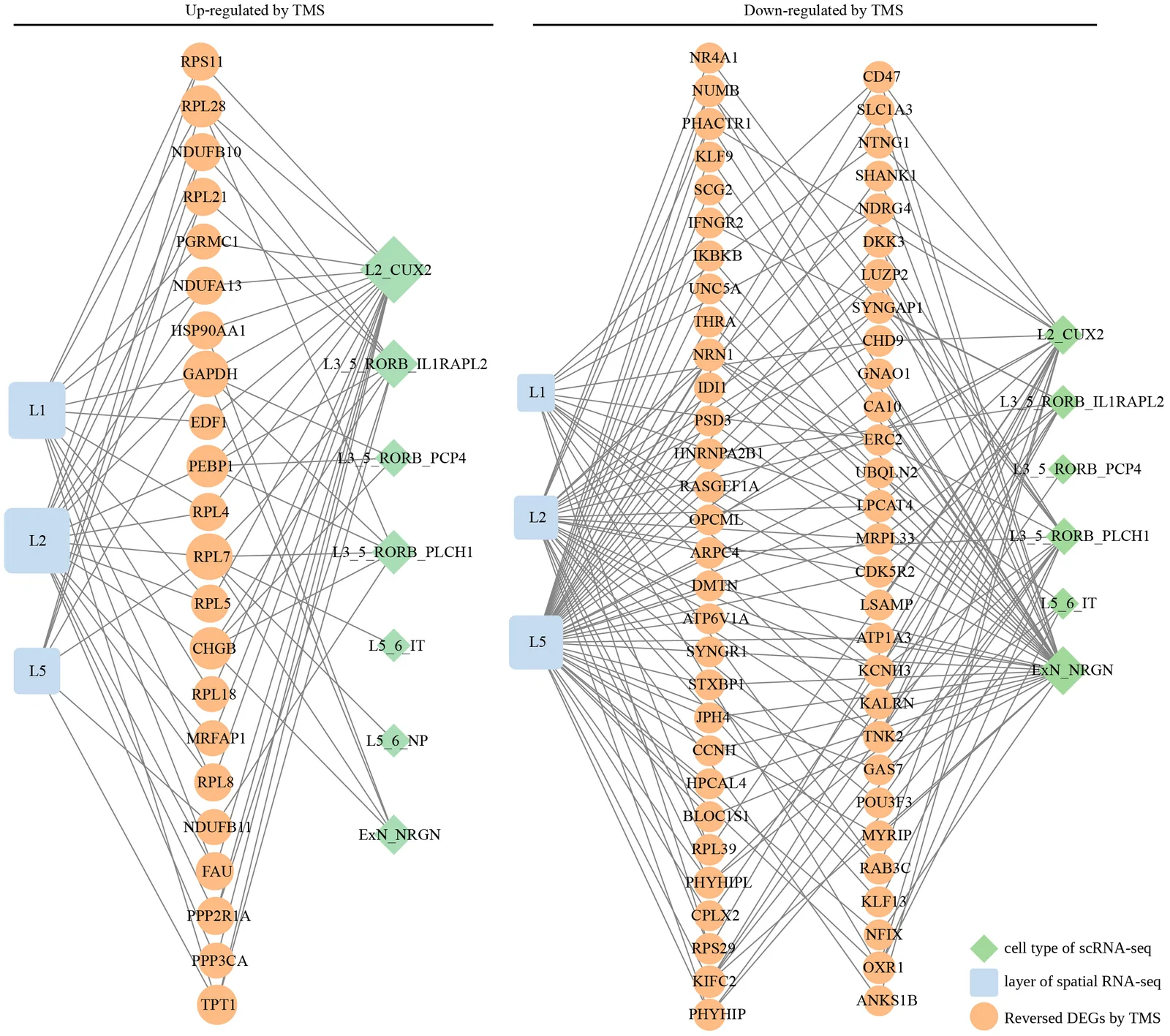
Exploratory integrative analysis of directional-overlap genes across cortical layers and excitatory neuronal subtypes. The left and right panels show genes up-regulated and down-regulated by TMS, respectively. Blue squares represent cortical layers from spatial RNA-seq (L1/L2/L5); green diamonds represent excitatory neuronal subtypes from scRNA-seq (e.g., CUX2, RORB, IT, NRGN); orange circles represent differentially expressed genes with a direction of change opposite to that of TMS, shared between spatial RNA-seq and scRNA-seq.

### 3.3 Application of epigenomics

Evidence sources: clinical epigenetic studies in MDD and preclinical TMS studies; the TMS–lactate–lactylation pathway is proposed as a testable hypothesis. Epigenetic modification refers to alterations of the genome that do not change the DNA base-pair sequence and can arise as rapid responses to environmental signals (72). Since the concept was first proposed in 1942, the field of epigenetics has expanded substantially (73). Epigenetic modifications are involved in numerous developmental processes and disease states, including the pathophysiology of many neuropsychiatric disorders. Recent studies indicate that the biological effects of environmental factors in MDD and other stress-related disorders are mediated by multiple epigenetic mechanisms. These alterations can lead to abnormal neuroendocrine responses, impaired neuroplasticity, dysregulated neurotransmission, and glial dysfunction, all of which are implicated in the pathophysiology of MDD (74). Large-scale methylome-wide association studies (MWAS) have identified multiple methylation sites associated with MDD in blood and postmortem brain tissue (75–77). MWAS findings in blood and brain overlap substantially, with enrichment in expression quantitative trait loci (eQTLs) in blood and frontal cortex, CpG islands, and exons. These overlapping loci are also enriched in active chromatin states in the brain, including gene enhancers and active transcription start sites. Cell type-specific methylome-wide studies have identified multiple reproducible neuron- and glia-related signals across different brain tissue samples and in physically sorted nuclei (76). Cell type-specific analyses in blood have also identified significant methylome-wide associations in T cells, monocytes, and whole blood (76). N6-methyladenosine (m6A) methylation regulates gene and protein expression by influencing multiple aspects of messenger RNA (mRNA) metabolism (78, 79). Summary data-based Mendelian randomization (SMR) analysis has identified three m6A sites associated with MDD (78). In chronic stress mouse models, 5-Hz rTMS promotes gene silencing by enhancing histone H3 methylation in the frontal cortex (FC) and DNA methylation in mature dentate gyrus (DG) neurons, thereby facilitating dendritic and synaptic remodeling in the FC and hippocampus and activating the corticolimbic system (48). Genome-scale studies characterizing DNA methylation (DNAm) patterns associated with TMS treatment response remain scarce. Existing evidence nonetheless suggests that rTMS can induce specific epigenetic marks that may regulate downstream processes and gene expression, thereby partly explaining its antidepressant effects.

Lactylation is a newly recognized post-translational protein modification that targets lysine residues in both histone and nonhistone proteins (80). Studies have shown that protein lactylation is involved in neurodevelopment, synapse formation, inflammatory and immune regulation, and tumor migration and invasion (81, 82). Lactylation is widely present in brain cells, including glutamatergic neurons, gamma-aminobutyric acid (GABA)-ergic neurons, astrocytes, and microglia (83). By increasing histone lactylation, lactate can alter gene expression programs and play a crucial role in brain development, aging, and disease (81). For example, exercise-induced lactate promotes the conversion of microglia from a proinflammatory to an anti-inflammatory phenotype through histone lactylation, thereby preventing cognitive impairment (84). In PD, elevated lactate levels accelerate histone 3 lysine 9 lactylation (H3K9la) at the solute carrier family 7 member 11 (SLC7A11) promoter, thereby promoting microglial activation and neuroinflammation (81). More than 200 proteins in hippocampal and cortical neurons contain lactylation sites associated with neural regeneration, axonal regeneration, and neuronal projection (82). Lactate can also influence neurodevelopment and neurological disease by increasing nonhistone protein lactylation. For example, histone deacetylase 6 (HDAC6) promotes α-tubulin lactylation in a lactate concentration-dependent manner, thereby participating in neurite outgrowth and branching (82). Clinical studies have shown that low-frequency rTMS decreases motor cortical excitability, whereas high-frequency rTMS increases it; both are accompanied by significant increases in blood lactate (85). These findings suggest a hypothetical pathway in which TMS-induced increases in blood lactate could enhance histone/protein lactylation and thereby modulate neuronal and glial function. We emphasize that peripheral lactate elevation does not by itself demonstrate increased brain lactate or TMS-induced lactylation; this pathway therefore requires direct experimental testing.

### 3.4 Application of proteomics

Evidence sources: proteomic studies in MDD postmortem brain tissue and exploratory integration with TMS transcriptomic data (hypothesis-generating). Proteomics has made substantial progress in psychiatric research in recent years, particularly in biomarker discovery, elucidation of pathological mechanisms, identification of therapeutic targets, and prediction of individualized treatment response. Qi et al. reported 180 and 258 differentially expressed proteins (DEPs) in the PFC and ACC, respectively, of patients with MDD compared with their corresponding control groups (86). However, a more recent study by Blaze et al. identified only 36 significantly altered proteins in the DLPFC of patients with MDD, including 2 downregulated and 34 upregulated proteins (FDR < 0.1 and |fold change [FC]| ≥ 2) (87) (**Figure 3A**). Another proteomic analysis of the ACC, using the same screening criteria, detected no differentially expressed proteins (88). By comparing the liquid chromatography-tandem mass spectrometry (LC-MS/MS) methods used across studies, we noted that Qi et al. employed nanoflow LC-MS/MS, which offers greater sensitivity than conventional LC-MS/MS and is better suited to analyzing protein/peptide mixtures when sample quantities are limited. These findings suggest that: (1) cortical samples from MDD may not exhibit major abnormalities at the level of the global proteome; and (2) the abundance and detectability of proteins/peptides are strongly influenced by methodological sensitivity and accuracy. Cross-dataset comparison identified LRSAM1, RAP1A, and CD9 as candidate molecules showing opposite directions of alteration between MDD proteomic data and TMS-related transcriptomic data (**Figure 3A**). In addition, proteomic profiling of extracellular vesicles (EVs) derived from the ACC revealed 70 differentially expressed proteins (48 upregulated and 22 downregulated) (88). A similar direction-aware comparison identified VAMP1, TUBA1C, PACSIN1, NCAM1, and DPYSL3 as additional candidate molecules (**Figure 3B**). These cross-platform overlaps are hypothesis-generating and do not demonstrate protein-level reversal by TMS in patients.

**Figure 3:**
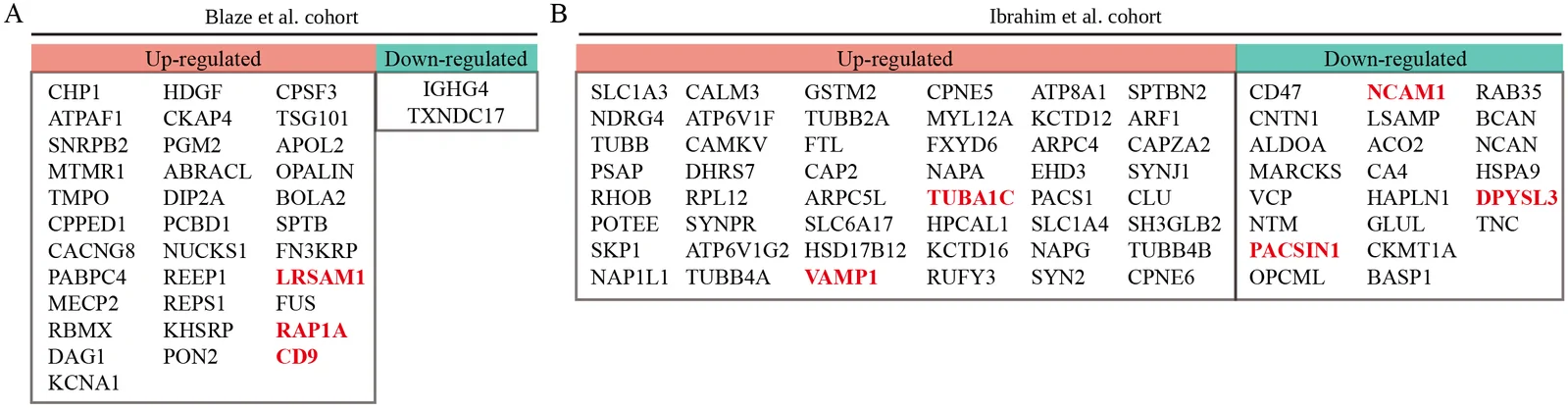
Integrative proteomic -transcriptomic analysis identifying candidate molecules with opposite directional changes between MDD proteomic and TMS-related transcriptomic datasets. **(A)** Blaze et al. cohort; LRSAM1, RAP1A and CD9 (red) were identified as candidate cross-dataset molecules showing opposite directional changes. **(B)** Ibrahim et al. cohort; a similar comparison identified VAMP1, TUBA1C, PACSIN1, NCAM1 and DPYSL3 (red) as additional candidate molecules. Genes highlighted in red are candidate molecules with opposite directional changes; only genes mappable to the cross-dataset comparison are shown.

### 3.5 Application of metabolomics

Evidence sources: metabolite inference from MDD single-cell transcriptomic data and in vitro/preclinical TMS studies; direct metabolomic evidence in TMS-treated TRD patients is limited. Exploratory scFEA-based inference from single-cell/nucleus transcriptomic data identified cell type-specific differences across multiple metabolic features in the PFC of patients with MDD. For example, exploratory cell-level signals involving glycine-, cholesterol-, and succinate-related metabolic features were observed in excitatory and inhibitory neurons (**Figure 4**). Because these comparisons were performed at the cell level without explicit modeling of donor dependence, the statistical significance may be affected by within-donor correlation; **Figure 4** should therefore be interpreted as hypothesis-generating rather than as confirmatory donor-level metabolic evidence. *In vitro* studies have shown that TMS can reduce neuronal levels of glycine, phenylalanine, isoleucine, serine, threonine, glycyl-glutamic acid, cholesterol, pyroglutamate, and succinate (89). Lipidomic studies further indicate that rTMS can alter the lipid composition of the hippocampus and PFC in rats exposed to chronic stress and may be more effective than fluoxetine in modulating hippocampal glycerophospholipids and sphingolipids (90). Regulation of the brain lipidome may therefore contribute to the antidepressant effects of rTMS. In addition, rTMS can alter neurotransmitter concentrations in cortical and subcortical regions of the rat brain, including glutamate, GABA, dopamine, and serotonin. Specifically, rTMS significantly decreases cortical 5-hydroxytryptamine turnover and hippocampal dopamine content, while significantly increasing striatal dopamine content. It also reduces hippocampal α-aminoadipic acid as well as striatal serine, threonine, sarcosine, aspartate, and glutamate concentrations (91). In patients with MDD, an exploratory study reported changes in the plasma metabolomic profile after intermittent theta-burst stimulation (92), and peripheral metabolomic changes after rTMS have also been described in other psychiatric populations (93), supporting the feasibility of peripheral metabolomic monitoring of TMS effects.

**Figure 4:**
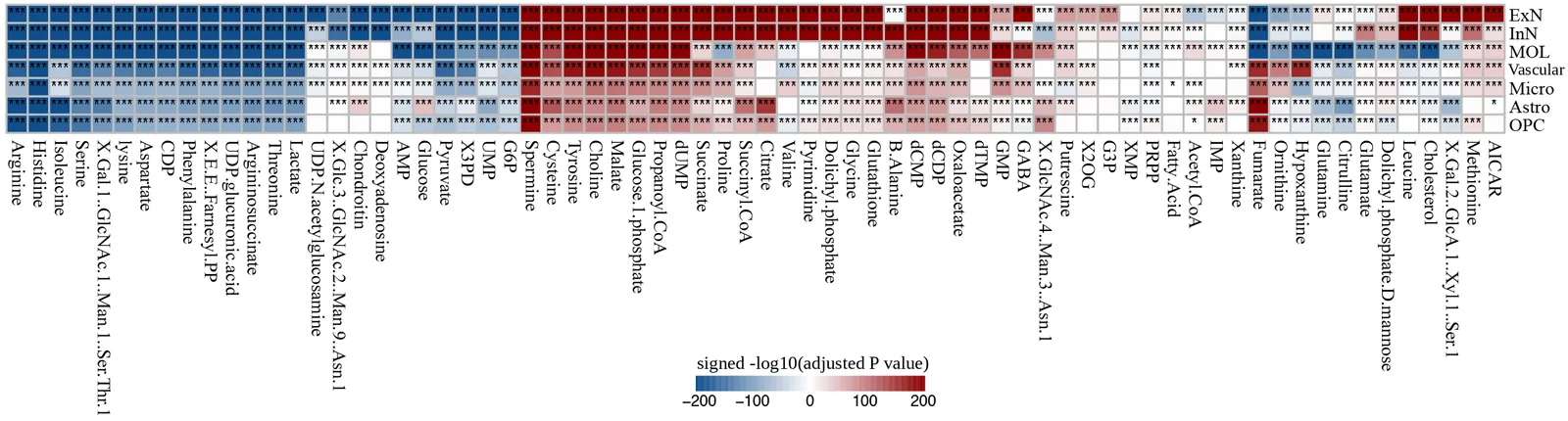
Exploratory cell type-specific metabolic signals in the PFC of patients with MDD inferred from single-cell transcriptomic data. Colour encodes the signed −log10(adjusted P value): red indicates relatively higher inferred metabolic signals in MDD and blue relatively lower signals. *P < 0.05, P < 0.01, *P < 0.001. Seven cell types are shown (ExN, InN, MOL, Vascular, Micro, Astro, OPC). Signals related to glycine, cholesterol and succinate were observed across excitatory and inhibitory neuronal populations. Findings are transcriptome-derived and hypothesis-generating.

### 3.6 Application of connectomics

Evidence sources: direct clinical imaging evidence in TRD and clinical imaging evidence from broader MDD populations. Imaging-guided TMS can support individualized target localization, treatment planning, dynamic network analysis, and prediction of therapeutic response. Owing to the noninvasive nature and clinical availability of neuroimaging, connectomic studies currently provide a larger body of direct patient evidence than the molecular omics domains. MDD is characterized by abnormalities within and between large-scale networks involved in cognitive control, attention, and self-referential processing (94). In patients with TRD, rTMS can induce widespread changes in functional connectivity, and treatment-related connectivity changes have been associated with symptom improvement. These observations support the potential use of functional and structural connectivity measures for target optimization and response prediction, while findings derived from broader MDD samples should not be interpreted as direct evidence in TRD.

#### 3.6.1 Target engagement and network propagation

A key principle emerging from connectomic studies is that TMS engages not only the stimulated site but also distributed, functionally connected regions: stimulation of the left prefrontal cortex enhances activity at the stimulation site and in connected limbic regions, including the bilateral medial PFC, right orbitofrontal cortex, left hippocampus, mediodorsal thalamic nucleus, bilateral putamen, pulvinar, and insula, while reducing activity in the right ventromedial PFC (95). The most influential connectivity-based model concerns the DLPFC–sgACC circuit: patients with stronger negative intrinsic functional connectivity between the left DLPFC (the conventional stimulation site) and the subgenual ACC (sgACC) show better clinical outcomes, a finding that has been reproduced across cohorts (96, 97). Acute TMS modulates connectivity within the cognitive control network and the default mode network (DMN), and in a study combining TMS with simultaneous fMRI and EEG, baseline TMS-evoked responses within cognitive control and limbic networks predicted clinical improvement, with efficacy associated with TMS-modulated connectivity between the left DLPFC and the posterior sgACC (98). However, the predictive utility of sgACC-based targeting is limited: in a large representative sample of patients with MDD, sgACC–stimulation-site functional connectivity explained only about 3% of the variance in treatment outcome (99). A core network model (CNM) based on sgACC/DLPFC connectivity improved within-subject reproducibility fivefold relative to sgACC connectivity alone and was associated with depression severity and TMS efficacy, although the optimal connectivity metrics remain to be established (100). Beyond the DLPFC–sgACC circuit, the locus coeruleus (LC) has emerged as a relevant propagation node: after accelerated high-frequency rTMS (aHF-rTMS), greater reductions in LC functional connectivity were associated with greater clinical improvement, with decreased connectivity between the LC and the superior frontal gyrus, precentral gyrus, middle occipital gyrus, and cerebellum (101). These findings emphasize the role of the LC in coordinating higher-order cognitive functions through its widespread prefrontal connections and suggest that LC–cerebellar connectivity may serve as a predictive marker of aHF-rTMS efficacy.

#### 3.6.2 Functional network reorganization

At the network level, normalization of depression-related dysfunction within and between large-scale functional networks—including the DMN, central executive network (CEN), and salience network (SN)—is associated with improvement in depressive symptoms (102, 103). rTMS induces widespread, acute, and transient changes in functional connectivity in patients with TRD, which account for roughly 30% of the variance in improvement on the Montgomery–Åsberg Depression Rating Scale, with the strongest predictive associations involving connectivity between prefrontal regions and motor, parietal, and insular cortices, as well as bilateral thalamic connectivity (104). Symptom improvement after TMS is associated with reduced connectivity between the sgACC and the DMN, left DLPFC, and insula, as well as reduced connectivity between the hippocampus and the SN (105). An iTBS study showed no significant changes in functional connectivity between individualized stimulation targets and either the CEN or dorsal cingulate cortex, but active iTBS increased the magnitude of negative functional connectivity between the target and the DMN, an effect that persisted at 1-month follow-up and correlated with symptom improvement (106). A study of low-frequency right-sided rTMS (LFR) showed that LFR increased functional connectivity between the left sgACC and parietal regions of the ventral attention network, suggesting that the antidepressant mechanism of low-frequency right-sided stimulation may differ from that of high-frequency left-sided rTMS. Stratification by a cognitive biotype of depression—characterized by treatment resistance, impaired cognitive control, and dysfunction in cognitive control circuits involving the dorsolateral PFC (dlPFC) and dorsal ACC (dACC)—showed that TMS-related improvements in cognitive control circuitry occurred only in individuals with low baseline dlPFC–dACC connectivity (107). Finally, personalized continuous gamma-rhythm inhibitory TMS based on effective connectivity from the right anterior insula to the left DLPFC showed efficacy comparable to that of conventional rTMS in patients with depression (8). Overall, connectivity between the DLPFC and ACC represents a promising diagnostic and response biomarker, but the optimal method for calculating functional connectivity remains to be determined, and prospective validation at the individual level is still needed.

#### 3.6.3 Structural connectivity and plasticity

Structural imaging complements functional connectivity findings. The amygdala—a key structure determining physiological, emotional, mnemonic, and behavioral responses to affective stimuli (108–110)—shows abnormal activity in depression and anxiety disorders (111, 112); six weeks of TMS increased overall amygdala volume and the volume of the stimulated left DLPFC in adolescents with TRD (113, 114). TMS of the ventrolateral PFC (vlPFC) produces acute, focal modulation of amygdala blood oxygenation level-dependent activity, and the magnitude of this effect is proportional to fiber density in the vlPFC–amygdala white-matter pathway, supporting the notion that cortico-subcortical structural connectivity facilitates stimulation-induced communication (115). White-matter integrity itself is modifiable by TMS: after 4–6 weeks of daily left-sided rTMS, mean fractional anisotropy (FA) in left prefrontal white matter increased without evidence of white-matter damage on the stimulated side (116); in patients with TRD, reduced FA in the left middle frontal gyrus improved after active rTMS in association with symptom alleviation (117); and FA in the anterior thalamic radiations is associated with symptom improvement in patients with MDD (118). Animal studies further suggest that rTMS may ameliorate chronic stress-related white-matter alterations linked to brain maturation, including changes in the corpus callosum, fornix, external capsule, and internal capsule, possibly through enhanced myelination (119).

#### 3.6.4 Other imaging modalities and translational implications

Additional modalities provide convergent evidence. Magnetic resonance spectroscopy (MRS) analyses have shown that, compared with nonresponders, responders have lower baseline glutamate concentrations in the DLPFC, followed by increased glutamate concentrations after successful rTMS treatment; these glutamate changes correlate with clinical improvement and interact with stimulation intensity (120). PET studies demonstrate that rTMS suppresses glucose metabolism in the left temporal cortex and fusiform gyrus of patients with TRD while enhancing glucose uptake in the medial PFC and ACC (121), and distinct frontal-temporal-limbic metabolic patterns differentiate responders from nonresponders, with preserved orbitofrontal volume and amygdala metabolism proposed as prerequisites for a favorable response (122). A multi-omics analysis integrating TMS-EEG with RNA-seq and scRNA-seq data further suggests that reduced signal propagation from the left dlPFC to the salience network in patients with TRD is associated with oligodendrocyte-specific gene expression (123), providing a concrete example of how molecular and systems-level observations may converge. Collectively, these connectomic and neurometabolic findings support the use of imaging measures for target optimization, response prediction, and individualized treatment planning in TMS for TRD.

## 4 Discussion

### 4.1 Integration across omics levels

Although each omics level provides a distinct perspective on the mechanisms of TMS, the relationships among molecular, cellular, and circuit-level findings remain largely unexplored, and a linear molecular-to-circuit cascade should not be assumed where direct evidence is lacking. The evidence reviewed here supports intra-level effects at each omics level: TMS modulates cell type-specific transcriptional programs (Section 3.1), region- and subtype-specific spatial transcriptomic patterns (Section 3.2), epigenetic marks such as DNA methylation and histone lactylation (Section 3.3), the expression of synapse-related proteins (Section 3.4), and neurotransmitter and metabolite dynamics (Section 3.5), while connectomic studies demonstrate reproducible effects on distributed brain networks (Section 3.6; summarized in **Table 1**). By contrast, direct evidence linking these levels—for example, demonstrating that a TMS-induced molecular change in a defined cell population causes a specific network-level change in the same individual—is currently absent and constitutes a major knowledge gap (**Figure 5**). Proposed cross-level links, such as the association between TMS-evoked propagation from the left DLPFC to the salience network and oligodendrocyte-related gene expression (123), or between peripheral lactate elevation and histone lactylation (85), remain correlational or indirect and require targeted experimental testing. We therefore regard the identification of validated cross-level relationships as the central challenge for the field.

**Table 1:** Summary of multi-omics evidence on the mechanisms of TMS in TRD and clinical implications.

Omics level	Key findings	Evidence source and level of directness	Clinical implications
Transcriptomics (bulk RNA-seq, scRNA-seq)	TMS is associated with cell type- and region-specific transcriptional changes; cross-dataset analyses identify DDX17, WSB1, and DCLK1 as candidate TMS-related genes linked to neuronal development and plasticity.	Preliminary to moderate; primarily cross-species and cross-dataset, hypothesis-generating evidence	Candidate molecular targets and biological hypotheses for protocol optimization
Spatial transcriptomics	Preclinical studies indicate cortical layer- and region-specific transcriptional responses to TMS.	Preliminary; preclinical evidence	May help refine mechanistic understanding of spatially specific stimulation effects
Epigenomics	Preclinical evidence supports TMS-related DNA and histone methylation changes; lactylation represents a proposed mechanism requiring direct validation.	Preliminary	Potential epigenetic biomarkers and therapeutic targets for future validation
Proteomics	MDD proteomic datasets and direction-aware comparison with TMS transcriptomic data identify candidate synapse-related molecules, including LRSAM1 and NCAM1.	Preliminary and indirect	Candidate protein biomarkers requiring prospective validation in TMS-treated TRD
Metabolomics	Preclinical and exploratory clinical evidence suggests changes in neurotransmitters, lipid metabolism, and peripheral metabolites; single-cell metabolic inference identifies cell type-specific metabolic abnormalities in MDD.	Preliminary	Potential treatment-monitoring and stratification markers after prospective validation
Connectomics (fMRI, DTI and related imaging)	Clinical studies show TMS-related changes in DLPFC-sgACC and other network connections, large-scale network organization, and white-matter measures; several connectivity metrics are associated with response.	Moderate to strong for selected clinical imaging findings; evidence varies by measure and population	Individualized targeting, response prediction, and imaging-guided treatment planning

**Figure 5:**
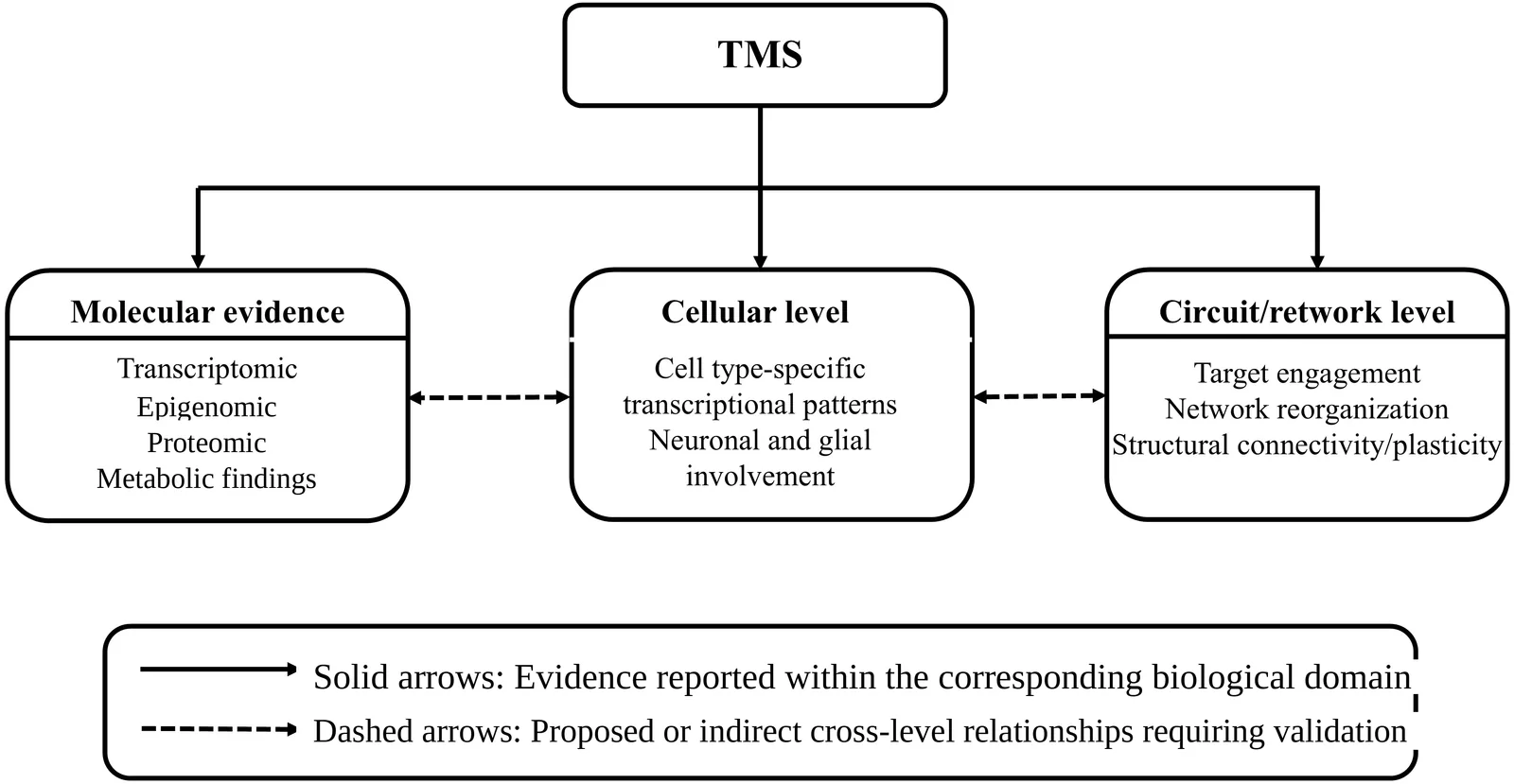
Integrative framework of multi-omics evidence on TMS in TRD. The figure summarizes findings across molecular (transcriptomic, epigenomic, proteomic and metabolic findings), cellular (cell type-specific transcriptional patterns and neuronal/glial involvement) and circuit/network (target engagement, network reorganization, structural connectivity/plasticity) levels. Solid arrows indicate evidence reported within the corresponding biological domain, whereas dashed arrows indicate proposed or indirect cross-level relationships requiring further validation. The framework does not imply a continuous molecular-to-cellular-to-circuit causal cascade.

### 4.2 Clinical implications and future directions

From a clinical perspective, the multi-omics evidence summarized here suggests three potential implications. First, connectomic findings support further development of connectivity-based targeting, particularly the identification of DLPFC subregions according to their functional relationship with the sgACC, as a strategy for individualizing stimulation sites. Second, molecular correlates of treatment response, including synapse-related proteins and peripheral metabolites, may serve as candidate biomarkers for predicting or monitoring therapeutic response after prospective validation and could eventually support earlier treatment adjustment in nonresponders. Third, the cell type- and region-specific transcriptional effects of TMS suggest that future protocol optimization may benefit from considering biological targets in addition to clinical symptoms. Nevertheless, most molecular evidence currently derives from preclinical, cross-sectional, or cross-dataset studies. Prospective longitudinal studies that measure molecular and circuit-level changes in the same patients with TRD are therefore needed before these findings can be translated into routine clinical practice.

### 4.3 Limitations

Several limitations of this review should be acknowledged. First, the evidence base is heterogeneous: much of the mechanistic evidence derives from broader MDD populations, animal models, or indirect experimental systems rather than from TMS-treated TRD patients, and the source and level of directness of the evidence were therefore explicitly distinguished throughout Sections 3.1–3.6. Second, the secondary analyses presented in **Figures 1**–**4** are exploratory and hypothesis-generating. In particular, the single-cell/nucleus differential-expression analyses and scFEA comparisons were performed at the cell level without explicit donor-level modeling; therefore, within-donor dependence may have inflated statistical significance, and these findings should not be interpreted as confirmatory subject-level associations. In addition, these analyses integrate publicly available datasets obtained from different species, platforms, and processing pipelines, and the identified cross-dataset overlaps require independent validation. Future confirmatory analyses should use donor-aware approaches, such as cell type-specific pseudobulk differential expression, mixed-effects models incorporating subject identity, or donor-level summaries of inferred metabolic features. Third, the literature search was restricted to English-language publications and to the databases and search terms specified in Section 2.1. Fourth, the field is evolving rapidly, and conclusions may change as larger, longitudinal multi-omics studies in TMS-treated patients become available.

## 5 Conclusions

This review summarizes recent evidence on transcranial magnetic stimulation (TMS) in major depressive disorder (MDD) and treatment-resistant depression (TRD) across transcriptomic, epigenomic, proteomic, metabolomic, and connectomic levels. Current findings indicate that TMS is associated with biological changes across these levels, including cell type-specific transcriptional responses, epigenetic regulation, candidate protein and metabolic changes, and remodeling of distributed brain networks. However, whether and how these changes are mechanistically linked across biological scales remains largely unresolved.

Important challenges remain, including small sample sizes, heterogeneous patient populations and stimulation protocols, reliance on animal models and indirect cross-dataset comparisons, and limited spatiotemporal resolution. Causal relationships between molecular alterations, circuit-level changes, and clinical outcomes have not been established in the same patients. Future longitudinal multi-omics studies in well-characterized TRD cohorts should integrate molecular biomarkers with stimulation parameters, target engagement, network changes, and clinical outcomes to identify reproducible response markers and support more individualized TMS treatment.

## References

[1] Fava M . The challenges of defining and managing treatment-resistant depression in research and practice. World Psychiatry. (2023) 22:350–1. doi: 10.1002/wps.21128 37713579 PMC10503900

[2] McIntyre RS Alsuwaidan M Baune BT Berk M Demyttenaere K Goldberg JF . Treatment-resistant depression: definition, prevalence, detection, management, and investigational interventions. World Psychiatry. (2023) 22:394–412. doi: 10.1002/wps.21120 37713549 PMC10503923

[3] Barker AT Jalinous R Freeston IL . Non-invasive magnetic stimulation of human motor cortex. Lancet. (1985) 1:1106–7. doi: 10.1016/s0140-6736(85)92413-4 2860322

[4] Siddiqi SH Schaper F Horn A Hsu J Padmanabhan JL Brodtmann A . Brain stimulation and brain lesions converge on common causal circuits in neuropsychiatric disease. Nat Hum Behav. (2021) 5:1707–16. doi: 10.1038/s41562-021-01161-1 34239076 PMC8688172

[5] van Rooij SJH Arulpragasam AR McDonald WM Philip NS . Accelerated TMS - moving quickly into the future of depression treatment. Neuropsychopharmacology. (2024) 49:128–37. 10.1038/s41386-023-01599-z PMC10700378 37217771

[6] Xing H Liu X Zhang Y Wu J Li M Ye H . Advances in repetitive transcranial magnetic stimulation in the treatment of treatment-resistant depression: a comprehensive review. Rev Neurosci. (2025). doi: 10.1515/revneuro-2024-0151 39946748

[7] Hflich G Kasper S Hufnagel A Ruhrmann S Mller H-J . Application of transcranial magnetic stimulation in treatment of drug-resistant major depression - a report of two cases. Hum Psychopharmacol - Clin Exp. (1993) 8(5):361–5.

[8] Morriss R Briley PM Webster L Abdelghani M Barber S Bates P . Connectivity-guided intermittent theta burst versus repetitive transcranial magnetic stimulation for treatment-resistant depression: a randomized controlled trial. Nat Med. (2024) 30:403–13. doi: 10.1038/s41591-023-02764-z 38228914 PMC10878976

[9] Cole J Sohn MN Harris AD Bray SL Patten SB McGirr A . Efficacy of adjunctive D-cycloserine to intermittent theta-burst stimulation for major depressive disorder: A randomized clinical trial. JAMA Psychiatry. (2022) 79:1153–61. 10.1001/jamapsychiatry.2022.3255 PMC9557938 36223114

[10] Sheline YI Makhoul W Batzdorf AS Nitchie FJ Lynch KG Cash R . Accelerated intermittent theta-burst stimulation and treatment-refractory bipolar depression: A randomized clinical trial. JAMA Psychiatry. (2024) 81:936–41. doi: 10.1001/jamapsychiatry.2024.1787 38985492 PMC11238064

[11] Bastiaens J Brown N Bermudes RA Juusola JL Bravata DM Marton TF . Utilization and outcomes of transcranial magnetic stimulation and usual care for MDD in a large group psychiatric practice. BMC Psychiatry. (2024) 24:497. doi: 10.1186/s12888-024-05928-4 38982458 PMC11234753

[12] Papakostas GI Trivedi MH Shelton RC Iosifescu DV Thase ME Jha MK . Comparative effectiveness research trial for antidepressant incomplete and non-responders with treatment resistant depression (ASCERTAIN-TRD) a randomized clinical trial. Mol Psychiatry. (2024) 29:2287–95. doi: 10.1038/s41380-024-02468-x 38454079 PMC11412904

[13] Cristancho M Fava M Ingelfinger J Nikayin S Sanacora G . Depression - advanced treatments for treatment-resistant depression. N Engl J Med. (2024) 390:e44. doi: 10.1056/nejmp2310181 38810184

[14] Dalhuisen I Bui K Kleijburg A van Oostrom I Spijker J van Exel E . Cost-effectiveness of rTMS as a next step in antidepressant non-responders: A randomized comparison with current antidepressant treatment approaches. Acta Psychiatr Scand. (2024) 151(5):613–24. doi: 10.1176/appi.ajp.20230556 39709996 PMC11962342

[15] Croarkin PE Zuckerman S Middleton VJ Monira N Kriske J Bowman J . Clinical outcomes in adolescents undergoing sequential bilateral 1 Hz/20 Hz transcranial magnetic stimulation for treatment resistant depression. Brain Stimul. (2024) 17:431–3. doi: 10.1016/j.brs.2024.03.018 38570160

[16] Robin A Thomas-Ollivier V Sauvaget A Pere M Bulteau S . Psychomotor retardation: What about the partial responders to magnetic transcranial stimulation in treatment resistant depression? J Psychiatr Res. (2024) 173:309–16. doi: 10.1016/j.jpsychires.2024.03.050 38569451

[17] Chopra A Singal P Kodya S . Impact of deep transcranial magnetic stimulation on insomnia outcomes in patients with treatment-resistant depression: a retrospective study. J Clin Sleep Med. (2024) 20:813–5. doi: 10.5664/jcsm.10954 38152858 PMC11063691

[18] Kweon J Fukuda AM Gobin AP Haq L Carpenter LL Brown JC . Effect of sleep quality on repetitive transcranial magnetic stimulation outcomes in depression. Front Psychiatry. (2024) 15:1458696. doi: 10.3389/fpsyt.2024.1458696 39376965 PMC11456523

[19] Göke K McClintock SM Mah L Rajji TK Lee HH Nestor SM . Cognitive profiles in treatment-resistant late-life depression and their impact on treatment outcomes. Biol Psychiatry Cognit Neurosci Neuroimaging. (2024) 9:1199–210. 10.1016/j.bpsc.2024.07.009 39053577

[20] Yıldız T Oğuzhanoğlu NK Topak OZ . Cognitive outcomes of transcranial magnetic stimulation in treatment-resistant depression: a randomized controlled study. Turk J Med Sci. (2023) 53:253–63. 10.55730/1300-0144.5580 PMC10387879 36945926

[21] Toffanin T Folesani F Ferrara M Belvederi Murri M Zerbinati L Caruso R . Cognitive functioning as predictor and marker of response to repetitive transcranial magnetic stimulation in depressive disorders: A systematic review. Gen Hosp Psychiatry. (2022) 79:19–32. doi: 10.1016/j.genhosppsych.2022.10.001 36240649

[22] Li J Li X Liu J Wei S Zhou D Wang D . Relationships between clinical symptoms, cognitive functioning, and TMS-evoked potential features in patients with major depressive disorder. Prog Neuro-Psychopharmacol Biol Psychiatry. (2025) 136:111184. doi: 10.1016/j.pnpbp.2024.111184 39550059

[23] Guo B Zhang M Hao W Wang Y Zhang T Liu C . Neuroinflammation mechanisms of neuromodulation therapies for anxiety and depression. Transl Psychiatry. (2023) 13:5. doi: 10.1038/s41398-022-02297-y 36624089 PMC9829236

[24] Tian L Sun SS Cui LB Wang SQ Peng ZW Tan QR . Repetitive transcranial magnetic stimulation elicits antidepressant- and anxiolytic-like effect via nuclear factor-E2-related factor 2-mediated anti-inflammation mechanism in rats. Neuroscience. (2020) 429:119–33. doi: 10.1016/j.neuroscience.2019.12.025 31918011

[25] Tateishi H Mizoguchi Y Kawaguchi A Imamura Y Matsushima J Kunitake H . Changes in interleukin-1 beta induced by rTMS are significantly correlated with partial improvement of cognitive dysfunction in treatment-resistant depression: a pilot study. Psychiatry Res. (2020) 289:112995. doi: 10.1016/j.psychres.2020.112995 32371273

[26] Shinba T Kariya N Matsuda S Matsuda H Obara Y . Increase of frontal cerebral blood volume during transcranial magnetic stimulation in depression is related to treatment effectiveness: A pilot study with near-infrared spectroscopy. Psychiatry Clin Neurosci. (2018) 72:602–10. doi: 10.1111/pcn.12680 29774621

[27] Tateishi H Setoyama D Kang D Matsushima J Kojima R Fujii Y . The changes in kynurenine metabolites induced by rTMS in treatment-resistant depression: A pilot study. J Psychiatr Res. (2021) 138:194–9. doi: 10.1016/j.jpsychires.2021.04.009 33865168

[28] Lazary J Elemery M Dome P Kiss S Gonda X Tombor L . Peripheral endocannabinoid serum level in association with repetitive transcranial magnetic stimulation (rTMS) treatment in patients with major depressive disorder. Sci Rep. (2021) 11:8867. doi: 10.1038/s41598-021-87840-5 33893327 PMC8065048

[29] Xue SS Xue F Ma QR Wang SQ Wang Y Tan QR . Repetitive high-frequency transcranial magnetic stimulation reverses depressive-like behaviors and protein expression at hippocampal synapses in chronic unpredictable stress-treated rats by enhancing endocannabinoid signaling. Pharmacol Biochem Behav. (2019) 184:172738. doi: 10.1016/j.pbb.2019.172738 31229467

[30] Keck ME Welt T Müller MB Erhardt A Ohl F Toschi N . Repetitive transcranial magnetic stimulation increases the release of dopamine in the mesolimbic and mesostriatal system. Neuropharmacology. (2002) 43:101–9. doi: 10.1016/s0028-3908(02)00069-2 12213264

[31] Zangen A Hyodo K . Transcranial magnetic stimulation induces increases in extracellular levels of dopamine and glutamate in the nucleus accumbens. Neuroreport. (2002) 13:2401–5. doi: 10.1097/00001756-200212200-00005 12499837

[32] Ben-Shachar D Belmaker RH Grisaru N Klein E . Transcranial magnetic stimulation induces alterations in brain monoamines. J Neural Transm (Vienna). (1997) 104:191–7. doi: 10.1007/bf01273180 9203081

[33] Lisanby SH Belmaker RH . Animal models of the mechanisms of action of repetitive transcranial magnetic stimulation (RTMS): comparisons with electroconvulsive shock (ECS). Depress Anxiety. (2000) 12:178–87. doi: 10.1002/1520-6394(2000)12:3<178::aid-da10>3.0.co;2-n 11126193

[34] Meshkat S Alnefeesi Y Jawad MY J DDV N BR Ceban F . Brain-Derived Neurotrophic Factor (BDNF) as a biomarker of treatment response in patients with Treatment Resistant Depression (TRD): A systematic review & meta-analysis. Psychiatry Res. (2022) 317:114857. doi: 10.1016/j.psychres.2022.114857 36194941

[35] Lee CW Wu HF Chu MC Chung YJ Mao WC Li CT . Mechanism of intermittent theta-burst stimulation in synaptic pathology in the prefrontal cortex in an antidepressant-resistant depression rat model. Cereb Cortex. (2021) 31:575–90. 10.1093/cercor/bhaa244 32901273

[36] Demiroz D Cicek IE Kurku H Eren I . Neurotrophic Factor Levels and Cognitive Functions before and after the Repetitive Transcranial Magnetic Stimulation in Treatment Resistant Depression. J Coll Physicians Surg Pak. (2022) 32:335–9. 10.29271/jcpsp.2022.03.335 35148586

[37] Valiuliene G Valiulis V Dapsys K Vitkeviciene A Gerulskis G Navakauskiene R . Brain stimulation effects on serum BDNF, VEGF, and TNFα in treatment-resistant psychiatric disorders. Eur J Neurosci. (2021) 53:3791–802. doi: 10.1111/ejn.15232 33861484

[38] Elemery M Kiss S Dome P Pogany L Faludi G Lazary J . Change of circulating vascular endothelial growth factor level and reduction of anhedonia are associated in patients with major depressive disorder treated with repetitive transcranial magnetic stimulation. Front Psychiatry. (2022) 13:806731. doi: 10.3389/fpsyt.2022.806731 35711587 PMC9193814

[39] Ueyama E Ukai S Ogawa A Yamamoto M Kawaguchi S Ishii R . Chronic repetitive transcranial magnetic stimulation increases hippocampal neurogenesis in rats. Psychiatry Clin Neurosci. (2011) 65:77–81. doi: 10.1111/j.1440-1819.2010.02170.x 21265939

[40] Wang HN Wang L Zhang RG Chen YC Liu L Gao F . Anti-depressive mechanism of repetitive transcranial magnetic stimulation in rat: the role of the endocannabinoid system. J Psychiatr Res. (2014) 51:79–87. doi: 10.1016/j.jpsychires.2014.01.004 24479995

[41] Feng SF Shi TY Fan Y Wang WN Chen YC Tan QR . Long-lasting effects of chronic rTMS to treat chronic rodent model of depression. Behav Brain Res. (2012) 232:245–51. doi: 10.1016/j.bbr.2012.04.019 22537774

[42] Yamasaki K Hasegawa T Takeda M . Serum level of soluble interleukin 6 receptor is a useful biomarker for identification of treatment-resistant major depressive disorder. Neuropsychopharmacol Rep. (2020) 40:130–7. doi: 10.1002/npr2.12100 32162496 PMC7722658

[43] Leblhuber F Geisler S Ehrlich D Steiner K Reibnegger G Fuchs D . Repetitive transcranial magnetic stimulation in the treatment of resistant depression: changes of specific neurotransmitter precursor amino acids. J Neural Transm (Vienna). (2021) 128:1225–31. doi: 10.1007/s00702-021-02363-7 34244826 PMC8321996

[44] Yilmaz NS Sen B Karadag RF Aslan S Ekmekci Ertek I Bozkurt A . A kynurenine pathway enzyme aminocarboxymuconate-semialdehyde decarboxylase may be involved in treatment-resistant depression, and baseline inflammation status of patients predicts treatment response: a pilot study. J Neural Transm (Vienna). (2022) 129:1513–26. doi: 10.1007/s00702-022-02553-x 36334154

[45] Ozkan BN Bozali K Boylu ME Velioglu HA Aktas S Kirpinar I . Altered blood parameters in "major depression" patients receiving repetitive transcranial magnetic stimulation (rTMS) therapy: a randomized case-control study. Transl Psychiatry. (2024) 14:264. doi: 10.1038/s41398-024-02942-8 38918365 PMC11199570

[46] Tasset I Drucker-Colín R Peña J Jimena I Montilla P Medina FJ . Antioxidant-like effects and protective action of transcranial magnetic stimulation in depression caused by olfactory bulbectomy. Neurochem Res. (2010) 35:1182–7. doi: 10.1007/s11064-010-0172-9 20428940

[47] Luan D Zhao MG Shi YC Li L Cao YJ Feng HX . Mechanisms of repetitive transcranial magnetic stimulation for anti-depression: Evidence from preclinical studies. World J Psychiatry. (2020) 10:223–33. doi: 10.5498/wjp.v10.i10.223 33134113 PMC7582130

[48] Meneses-San Juan D Lamas M Ramírez-Rodríguez GB . Repetitive transcranial magnetic stimulation reduces depressive-like behaviors, modifies dendritic plasticity, and generates global epigenetic changes in the frontal cortex and hippocampus in a rodent model of chronic stress. Cells. (2023) 12:2026. doi: 10.3390/cells12162062 37626872 PMC10453847

[49] Lee CW Chu MC Wu HF Chung YJ Hsieh TH Chang CY . Different synaptic mechanisms of intermittent and continuous theta-burst stimulations in a severe foot-shock induced and treatment-resistant depression in a rat model. Exp Neurol. (2023) 362:114338. doi: 10.1016/j.expneurol.2023.114338 36717014

[50] Long Z Du L Zhao J Wu S Zheng Q Lei X . Prediction on treatment improvement in depression with resting state connectivity: A coordinate-based meta-analysis. J Affect Disord. (2020) 276:62–8. doi: 10.1016/j.jad.2020.06.072 32697717

[51] Xiong LL He R Du RL Niu RZ Xue LL Chen L . A single-nucleus transcriptomic atlas reveals cellular and genetic characteristics of Alzheimer’s-like pathology in aging tree shrews. Medcomm. (2025) 6:e70114. doi: 10.1002/mco2.70114 40093585 PMC11910145

[52] Xiong LL Du RL Niu RZ Xue LL Chen L Huangfu LR . Single-cell RNA sequencing reveals peripheral immunological features in Parkinson's disease. NPJ Parkinsons Dis. (2024) 10:185. doi: 10.1038/s41531-024-00790-3 39366969 PMC11452393

[53] Sun Z Zhang B Zhou J Luo Y Zhu X Wang Y . Integrated single-cell RNA-seq and ATAC-seq reveals heterogeneous differentiation of CD4(+) naive T cell subsets is associated with response to antidepressant treatment in major depressive disorder. Adv Sci (Weinh). (2024) 11:e2308393. doi: 10.1002/advs.202308393 38867657 PMC11321657

[54] Nagy C Maitra M Tanti A Suderman M Théroux JF Davoli MA . Single-nucleus transcriptomics of the prefrontal cortex in major depressive disorder implicates oligodendrocyte precursor cells and excitatory neurons. Nat Neurosci. (2020) 23:771–81. doi: 10.1038/s41593-020-0621-y 32341540

[55] Ramírez-Rodríguez GB Meneses San-Juan D Rico-Becerra AI González-Olvera JJ Reyes-Galindo V . Repetitive transcranial magnetic stimulation and fluoxetine reverse depressive-like behavior but with differential effects on Olig2-positive cells in chronically stressed mice. Neuropharmacology. (2023) 236:109567. 10.1016/j.neuropharm.2023.109567 37209812

[56] Maitra M Mitsuhashi H Rahimian R Chawla A Yang J Fiori LM . Cell type specific transcriptomic differences in depression show similar patterns between males and females but implicate distinct cell types and genes. Nat Commun. (2023) 14:2912. doi: 10.1038/s41467-023-38530-5 37217515 PMC10203145

[57] Ong RCS Tang AD . Subthreshold repetitive transcranial magnetic stimulation induces cortical layer-, brain region-, and protocol-dependent neural plasticity. Sci Adv. (2025) 11:eado6705. doi: 10.1126/sciadv.ado6705 39772671 PMC11708880

[58] Weiler M Stieger KC Shroff K Klein JP Wood WH Zhang Y . Transcriptional changes in the rat brain induced by repetitive transcranial magnetic stimulation. Front Hum Neurosci. (2023) 17:1215291. doi: 10.3389/fnhum.2023.1215291 38021223 PMC10679736

[59] Hwang W Choi JK Bang MS Park WY Oh BM . Gene expression profile changes in the stimulated rat brain cortex after repetitive transcranial magnetic stimulation. Brain Neurorehabil. (2022) 15:e27. doi: 10.12786/bn.2022.15.e27 36742089 PMC9833481

[60] Seaby EG Godwin A Meyer-Dilhet G Clerc V Grand X Fletcher T . Monoallelic de novo variants in DDX17 cause a neurodevelopmental disorder. Brain. (2024). doi: 10.1093/brain/awae320 39405200 PMC11967813

[61] Xie F Shen B Luo Y Zhou H Xie Z Zhu S . Repetitive transcranial magnetic stimulation alleviates motor impairment in Parkinson's disease: association with peripheral inflammatory regulatory T-cells and SYT6. Mol Neurodegener. (2024) 19:80. doi: 10.1186/s13024-024-00770-4 39456006 PMC11515224

[62] Chou YH Hickey PT Sundman M Song AW Chen NK . Effects of repetitive transcranial magnetic stimulation on motor symptoms in Parkinson disease: a systematic review and meta-analysis. JAMA Neurol. (2015) 72:432–40. doi: 10.1001/jamaneurol.2014.4380 25686212 PMC4425190

[63] Weimer JM Anton ES . Doubling up on microtubule stabilizers: synergistic functions of doublecortin-like kinase and doublecortin in the developing cerebral cortex. Neuron. (2006) 49:3–4. doi: 10.1016/j.neuron.2005.12.016 16387632

[64] Deuel TA Liu JS Corbo JC Yoo SY Rorke-Adams LB Walsh CA . Genetic interactions between doublecortin and doublecortin-like kinase in neuronal migration and axon outgrowth. Neuron. (2006) 49:41–53. doi: 10.1016/j.neuron.2005.10.038 16387638

[65] Koizumi H Tanaka T Gleeson JG . Doublecortin-like kinase functions with doublecortin to mediate fiber tract decussation and neuronal migration. Neuron. (2006) 49:55–66. doi: 10.1016/j.neuron.2005.10.040 16387639

[66] Shin E Kashiwagi Y Kuriu T Iwasaki H Tanaka T Koizumi H . Doublecortin-like kinase enhances dendritic remodelling and negatively regulates synapse maturation. Nat Commun. (2013) 4:1440. doi: 10.1038/ncomms2443 23385585 PMC4017031

[67] Nawabi H Belin S Cartoni R Williams PR Wang C Latremolière A . Doublecortin-like kinases promote neuronal survival and induce growth cone reformation via distinct mechanisms. Neuron. (2015) 88:704–19. doi: 10.1016/j.neuron.2015.10.005 26526391 PMC10069300

[68] Le Hellard S Håvik B Espeseth T Breilid H Løvlie R Luciano M . Variants in doublecortin- and calmodulin kinase like 1, a gene up-regulated by BDNF, are associated with memory and general cognitive abilities. PLoS One. (2009) 4:e7534. doi: 10.1371/journal.pone.0007534 19844571 PMC2760101

[69] Kleim JA Chan S Pringle E Schallert K Procaccio V Jimenez R . BDNF val66met polymorphism is associated with modified experience-dependent plasticity in human motor cortex. Nat Neurosci. (2006) 9:735–7. doi: 10.1038/nn1699 16680163

[70] Fitzsimmons S Oostra E Postma TS van der Werf YD van den Heuvel OA . Repetitive transcranial magnetic stimulation-induced neuroplasticity and the treatment of psychiatric disorders: State of the evidence and future opportunities. Biol Psychiatry. (2024) 95:592–600. doi: 10.1016/j.biopsych.2023.11.016 38040046

[71] Zuo C Cao H Ding F Zhao J Huang Y Li G . Neuroprotective efficacy of different levels of high-frequency repetitive transcranial magnetic stimulation in mice with CUMS-induced depression: Involvement of the p11/BDNF/Homer1a signaling pathway. J Psychiatr Res. (2020) 125:152–63. doi: 10.1016/j.jpsychires.2020.03.018 32289652

[72] Flynn LT Gao WJ . DNA methylation and the opposing NMDAR dysfunction in schizophrenia and major depression disorders: a converging model for the therapeutic effects of psychedelic compounds in the treatment of psychiatric illness. Mol Psychiatry. (2023) 28:4553–67. doi: 10.1038/s41380-023-02235-4 37679470 PMC11034997

[73] Deichmann U . Epigenetics: The origins and evolution of a fashionable topic. Dev Biol. (2016) 416:249–54. doi: 10.1016/j.ydbio.2016.06.005 27291929

[74] Yuan M Yang B Rothschild G Mann JJ Sanford LD Tang X . Epigenetic regulation in major depression and other stress-related disorders: molecular mechanisms, clinical relevance and therapeutic potential. Signal Transduct Target Ther. (2023) 8:309. doi: 10.1038/s41392-023-01519-z 37644009 PMC10465587

[75] Aberg KA Dean B Shabalin AA Chan RF Han LKM Zhao M . Methylome-wide association findings for major depressive disorder overlap in blood and brain and replicate in independent brain samples. Mol Psychiatry. (2020) 25:1344–54. 10.1038/s41380-018-0247-6 PMC6428621 30242228

[76] Chan RF Turecki G Shabalin AA Guintivano J Zhao M Xie LY . Cell type-specific methylome-wide association studies implicate neurotrophin and innate immune signaling in major depressive disorder. Biol Psychiatry. (2020) 87:431–42. doi: 10.1016/j.biopsych.2019.10.014 31889537 PMC9933050

[77] Barbu MC Shen X Walker RM Howard DM Evans KL Whalley HC . Epigenetic prediction of major depressive disorder. Mol Psychiatry. (2021) 26:5112–23. 10.1038/s41380-020-0808-3 PMC8589651 32523041

[78] Liufu C Luo L Pang T Zheng H Yang L Lu L . Integration of multi-omics summary data reveals the role of N6-methyladenosine in neuropsychiatric disorders. Mol Psychiatry. (2024) 29:3141–50. doi: 10.1038/s41380-024-02574-w 38684796

[79] Liu S Xiu J Zhu C Meng K Li C Han R . Fat mass and obesity-associated protein regulates RNA methylation associated with depression-like behavior in mice. Nat Commun. (2021) 12:6937. doi: 10.1038/s41467-021-27044-7 34836959 PMC8626436

[80] Zhang D Tang Z Huang H Zhou G Cui C Weng Y . Metabolic regulation of gene expression by histone lactylation. Nature. (2019) 574:575–80. doi: 10.1038/s41586-019-1678-1 31645732 PMC6818755

[81] Nohesara S Abdolmaleky HM Thiagalingam S . Potential for new therapeutic approaches by targeting lactate and pH mediated epigenetic dysregulation in major mental diseases. Biomedicines. (2024) 12:457. doi: 10.3390/biomedicines12020457 38398057 PMC10887322

[82] Sun S Xu Z He L Shen Y Yan Y Lv X . Metabolic regulation of cytoskeleton functions by HDAC6-catalyzed α-tubulin lactylation. Nat Commun. (2024) 15:8377. doi: 10.1038/s41467-024-52729-0 39333081 PMC11437170

[83] Li R Yang Y Wang H Zhang T Duan F Wu K . Lactate and lactylation in the brain: Current progress and perspectives. Cell Mol Neurobiol. (2023) 43:2541–55. doi: 10.1007/s10571-023-01335-7 36928470 PMC11410153

[84] Han H Zhao Y Du J Wang S Yang X Li W . Exercise improves cognitive dysfunction and neuroinflammation in mice through histone H3 lactylation in microglia. Immun Ageing. (2023) 20:63. doi: 10.1186/s12979-023-00390-4 37978517 PMC10655345

[85] Alagona G Coco M Rapisarda G Costanzo E Maci T Restivo D . Changes of blood lactate levels after repetitive transcranial magnetic stimulation. Neurosci Lett. (2009) 450:111–3. doi: 10.1016/j.neulet.2008.11.064 19084051

[86] Qi YJ Lu YR Shi LG Demmers JAA Bezstarosti K Rijkers E . Distinct proteomic profiles in prefrontal subareas of elderly major depressive disorder and bipolar disorder patients. Transl Psychiatry. (2022) 12:275. doi: 10.1038/s41398-022-02040-7 35821008 PMC9276790

[87] Blaze J Chen S Heissel S Alwaseem H Landinez Macias MP Peter C . Altered tRNA expression profile associated with codon-specific proteomic changes in the suicide brain. Mol Psychiatry. (2025). doi: 10.1038/s41380-025-02891-8 39809846 PMC12185327

[88] Ibrahim P Mitsuhashi H Taylor L Cleyle J Mechawar N Nagy C . Altered proteomics in brain extracellular vesicles from depressed individuals who died by suicide implicates synaptic processes. Int J Neuropsychopharmacol. (2025). doi: 10.1093/ijnp/pyaf012 39989284 PMC12122421

[89] Hong I Garrett A Maker G Mullaney I Rodger J Etherington SJ . Repetitive low intensity magnetic field stimulation in a neuronal cell line: a metabolomics study. PeerJ. (2018) 6:e4501. doi: 10.7717/peerj.4501 PMC5853602 29576970

[90] Xue SS Zhou CH Xue F Liu L Cai YH Luo JF . The impact of repetitive transcranial magnetic stimulation and fluoxetine on the brain lipidome in a rat model of chronic unpredictable stress. Prog Neuro-Psychopharmacol Biol Psychiatry. (2020) 102:109946. doi: 10.1016/j.pnpbp.2020.109946 32325156

[91] Poh EZ Hahne D Moretti J Harvey AR Clarke MW Rodger J . Simultaneous quantification of dopamine, serotonin, their metabolites and amino acids by LC-MS/MS in mouse brain following repetitive transcranial magnetic stimulation. Neurochem Int. (2019) 131:104546. doi: 10.1016/j.neuint.2019.104546 31518601

[92] Luo X Zhou Y Yuan S Chen X Zhang B . The changes in metabolomics profile induced by intermittent theta burst stimulation in major depressive disorder: an exploratory study. BMC Psychiatry. (2023) 23:550. doi: 10.1186/s12888-023-05044-9 37516823 PMC10387200

[93] Su H Yang P Chen T Deng D Zhong N Jiang H . Metabolomics changes after rTMS intervention reveal potential peripheral biomarkers in methamphetamine dependence. Eur Neuropsychopharmacol. (2022) 56:80–8. doi: 10.1016/j.euroneuro.2021.12.006 34990999

[94] Ha T Jakimier K O'Sullivan S . The use of MRI and TMS in treatment-resistant depression: advances in pediatric applications. Brain Sci. (2025) 15:194. doi: 10.3390/brainsci15020194 40002526 PMC11853665

[95] Li X Nahas Z Kozel FA Anderson B Bohning DE George MS . Acute left prefrontal transcranial magnetic stimulation in depressed patients is associated with immediately increased activity in prefrontal cortical as well as subcortical regions. Biol Psychiatry. (2004) 55:882–90. doi: 10.1016/j.biopsych.2004.01.017 15110731

[96] Fox MD Buckner RL White MP Greicius MD Pascual-Leone A . Efficacy of transcranial magnetic stimulation targets for depression is related to intrinsic functional connectivity with the subgenual cingulate. Biol Psychiatry. (2012) 72:595–603. doi: 10.1016/j.biopsych.2012.04.028 22658708 PMC4120275

[97] Cash RFH Weigand A Zalesky A Siddiqi SH Downar J Fitzgerald PB . Using brain imaging to improve spatial targeting of transcranial magnetic stimulation for depression. Biol Psychiatry. (2021) 90:689–700. doi: 10.1016/j.biopsych.2020.05.033 32800379

[98] He H Sun X Doose J Faller J McIntosh JR Saber GT . TMS-induced modulation of brain networks and its associations to rTMS treatment for depression: a concurrent fMRI-EEG-TMS study. Brain Stimulation. (2025) 18:1955–65. doi: 10.1016/j.brs.2025.10.013 41109521 PMC12716231

[99] Elbau IG Lynch CJ Downar J Vila-Rodriguez F Power JD Solomonov N . Functional connectivity mapping for rTMS target selection in depression. Am J Psychiatry. (2023) 180:230–40. doi: 10.1176/appi.ajp.20220306 36855880 PMC11446248

[100] Raij TT Komulainen E Aydogan DB Pamilo S Isometsä E Raij T . Depression core network-based individualized targeting for transcranial magnetic stimulation. Brain Stimul. (2023) 16:619–27. doi: 10.1016/j.brs.2023.03.005 36931462 PMC10404717

[101] Wu GR Baeken C . Exploring potential working mechanisms of accelerated HF-rTMS in refractory major depression with a focus on locus coeruleus connectivity. Eur Psychiatry. (2024) 67:e70. doi: 10.1192/j.eurpsy.2024.1769 39417327 PMC11730058

[102] Anderson RJ Hoy KE Daskalakis ZJ Fitzgerald PB . Repetitive transcranial magnetic stimulation for treatment resistant depression: re-establishing connections. Clin Neurophysiol. (2016) 127:3394–405. doi: 10.1016/j.clinph.2016.08.015 27672727

[103] Liston C Chen AC Zebley BD Drysdale AT Gordon R Leuchter B . Default mode network mechanisms of transcranial magnetic stimulation in depression. Biol Psychiatry. (2014) 76:517–26. doi: 10.1016/j.biopsych.2014.01.023 24629537 PMC4209727

[104] Ge R Humaira A Gregory E Alamian G MacMillan EL Barlow L . Predictive value of acute neuroplastic response to rTMS in treatment outcome in depression: a concurrent TMS-fMRI trial. Am J Psychiatry. (2022) 179:500–8. doi: 10.1176/appi.ajp.21050541 35582784

[105] Philip NS Barredo J van 't Wout-Frank M Tyrka AR Price LH Carpenter LL . Network mechanisms of clinical response to transcranial magnetic stimulation in posttraumatic stress disorder and major depressive disorder. Biol Psychiatry. (2018) 83:263–72. doi: 10.1016/j.biopsych.2017.07.021 28886760 PMC6679924

[106] Gajawelli N Geoly AD Batail JM Xiao X Maron-Katz A Cole E . Increased anti-correlation between the left dorsolateral prefrontal cortex and the default mode network following Stanford Neuromodulation Therapy (SNT): analysis of a double-blinded, randomized, sham-controlled trial. NPJ Ment Health Res. (2024) 3:35. doi: 10.1038/s44184-024-00073-y 38971869 PMC11227523

[107] Tozzi L Bertrand C Hack LM Lyons T Olmsted AM Rajasekharan D . A cognitive neural circuit biotype of depression showing functional and behavioral improvement after transcranial magnetic stimulation in the B-SMART-fMRI trial. Nat Ment Health. (2024) 2:987–98. doi: 10.1038/s44220-024-00271-9 39911692 PMC11798407

[108] Gründemann J Bitterman Y Lu T Krabbe S Grewe BF Schnitzer MJ . Amygdala ensembles encode behavioral states. Science. (2019) 364:eaav3932. 10.1126/science.aav8736 31000636

[109] Lesuis SL Park S Hoorn A Rashid AJ Mocle AJ Salter EW . Stress disrupts engram ensembles in lateral amygdala to generalize threat memory in mice. Cell. (2025) 188:121–140.e20. doi: 10.1016/j.cell.2024.10.034 39549697 PMC11726195

[110] Santoni G Astori S Leleu M Glauser L Zamora SA Schioppa M . Chromatin plasticity predetermines neuronal eligibility for memory trace formation. Science. (2024) 385:eadg9982. doi: 10.1126/science.adg9982 39052786

[111] Adhikari A Lerner TN Finkelstein J Pak S Jennings JH Davidson TJ . Basomedial amygdala mediates top-down control of anxiety and fear. Nature. (2015) 527:179–85. doi: 10.1038/nature15698 26536109 PMC4780260

[112] Yu XD Zhu Y Sun QX Deng F Wan J Zheng D . Distinct serotonergic pathways to the amygdala underlie separate behavioral features of anxiety. Nat Neurosci. (2022) 25:1651–63. doi: 10.1038/s41593-022-01200-8 36446933

[113] Seewoo BJ Rodger J Demitrack MA Heart KL Port JD Strawn JR . Neurostructural differences in adolescents with treatment-resistant depression and treatment effects of transcranial magnetic stimulation. Int J Neuropsychopharmacol. (2022) 25:619–30. doi: 10.1093/ijnp/pyac007 35089358 PMC9380715

[114] Kaiser RH Andrews-Hanna JR Wager TD Pizzagalli DA . Large-scale network dysfunction in major depressive disorder: a meta-analysis of resting-state functional connectivity. JAMA Psychiatry. (2015) 72:603–11. doi: 10.1001/jamapsychiatry.2015.0071 25785575 PMC4456260

[115] Sydnor VJ Cieslak M Duprat R Deluisi J Flounders MW Long H . Cortical-subcortical structural connections support transcranial magnetic stimulation engagement of the amygdala. Sci Adv. (2022) 8:eabn5803. doi: 10.1126/sciadv.abn5803 35731882 PMC9217085

[116] Kozel FA Johnson KA Nahas Z Nakonezny PA Morgan PS Anderson BS . Fractional anisotropy changes after several weeks of daily left high-frequency repetitive transcranial magnetic stimulation of the prefrontal cortex to treat major depression. J ECT. (2011) 27:5–10. doi: 10.1097/yct.0b013e3181e6317d 20559144 PMC2975808

[117] Peng H Zheng H Li L Liu J Zhang Y Shan B . High-frequency rTMS treatment increases white matter FA in the left middle frontal gyrus in young patients with treatment-resistant depression. J Affect Disord. (2012) 136:249–57. doi: 10.1016/j.jad.2011.12.006 22217432

[118] Barredo J Bellone JA Edwards M Carpenter LL Correia S Philip NS . White matter integrity and functional predictors of response to repetitive transcranial magnetic stimulation for posttraumatic stress disorder and major depression. Depress Anxiety. (2019) 36:1047–57. doi: 10.1002/da.22952 31475432 PMC8015421

[119] Chen J Li C Hong J Zhao F Zhang J Yang M . High-frequency repetitive transcranial magnetic stimulation enhances white matter integrity in a rat model of ischemic stroke: a diffusion tensor imaging study using tract-based spatial statistics. NeuroImage. (2025) 311:121204. doi: 10.1016/j.neuroimage.2025.121204 40222496

[120] Luborzewski A Schubert F Seifert F Danker-Hopfe H Brakemeier EL Schlattmann P . Metabolic alterations in the dorsolateral prefrontal cortex after treatment with high-frequency repetitive transcranial magnetic stimulation in patients with unipolar major depression. J Psychiatr Res. (2007) 41:606–15. doi: 10.1016/j.jpsychires.2006.02.003 16600298

[121] Li CT Wang SJ Hirvonen J Hsieh JC Bai YM Hong CJ . Antidepressant mechanism of add-on repetitive transcranial magnetic stimulation in medication-resistant depression using cerebral glucose metabolism. J Affect Disord. (2010) 127:219–29. doi: 10.1016/j.jad.2010.05.028 20598753

[122] Paillère Martinot ML Martinot JL Ringuenet D Galinowski A Gallarda T Bellivier F . Baseline brain metabolism in resistant depression and response to transcranial magnetic stimulation. Neuropsychopharmacology. (2011) 36:2710–9. 10.1038/npp.2011.161 PMC3230494 21849980

[123] Wada M Nakajima S Honda S Takano M Taniguchi K Tsugawa S . Reduced signal propagation elicited by frontal transcranial magnetic stimulation is associated with oligodendrocyte abnormalities in treatment-resistant depression. J Psychiatry Neurosci. (2022) 47:E325–35. doi: 10.1503/jpn.220102 36104082 PMC9484613

