# Supplementary material for: The application of transcranial magnetic stimulation in treatment-resistant depression: insights from omics
Source: Front Psychiatry. 2026 Oct 20;17:1938107. doi: 10.3389/fpsyt.2026.1938107 (PMC13729381; doi:10.3389/fpsyt.2026.1938107)
Supplement: Supplementary file 1 [file DataSheet1.docx]

**Supplementary Methods**

*Secondary analyses of publicly available omics datasets (supporting Figures 1–4)*

**1. Analysis of single-cell transcriptomic datasets from MDD patients**

Publicly available single-cell/nucleus RNA-sequencing datasets of postmortem brain tissues from patients with major depressive disorder (MDD) and healthy controls (HCs) were obtained from the Gene Expression Omnibus (GEO) under accession numbers GSE144136 and GSE213982. These datasets were reanalyzed to characterize cell-type-specific transcriptomic alterations associated with MDD and to compare these alterations with transcriptional changes induced by transcranial magnetic stimulation (TMS).

**2. Data acquisition, preprocessing, and quality control**

For datasets for which raw sequencing reads were reprocessed, raw sequencing data were processed using Cell Ranger v7.0.1 (10x Genomics) to generate gene-by-cell unique molecular identifier (UMI) count matrices. Subsequent analyses were performed using Seurat v4.2.2 in R. Cells/nuclei with fewer than 200 detected genes or with mitochondrial transcript proportions greater than 5% were excluded. Doublets were identified independently within each dataset using DoubletFinder v2.0.3 and removed before dataset integration. Following integration and clustering, clusters characterized by low numbers of detected genes, high mitochondrial transcript proportions, or absence of biologically interpretable cell-type marker expression were excluded from subsequent analyses.

**3. Integration and clustering of MDD single-cell transcriptomic datasets**

The GSE144136 and GSE213982 datasets were integrated using the reciprocal principal component analysis (RPCA) workflow implemented in Seurat v4.2.2. Briefly, individual datasets were normalized and subjected to principal component analysis independently. Integration anchors were identified using FindIntegrationAnchors with RPCA as the reduction method, and batch-corrected expression values were generated using IntegrateData. RPCA was selected because it identifies mutual nearest-neighbor anchors after reciprocal projection of datasets into each other's PCA space and is suitable for integrating datasets containing shared major cell populations while reducing overcorrection of biologically meaningful differences.

The integrated dataset was subsequently subjected to PCA-based dimensionality reduction. Cell-cell nearest-neighbor graphs were constructed using FindNeighbors, and unsupervised clustering was performed using FindClusters. Uniform manifold approximation and projection (UMAP) was used for visualization of the integrated cell populations.

**4. Cell-type annotation**

Cell identities were initially inferred using SingleR based on similarity between individual transcriptomic profiles and reference expression datasets. Automated annotations were subsequently manually examined and refined according to the expression of established canonical marker genes and the original annotations of the corresponding datasets.

Major cell populations were identified using the following representative markers: neurons, RBFOX1, SNAP25, and SYT1; excitatory neurons (ExN), SLC17A7 and CAMK2A; inhibitory neurons (InN), GAD1 and GAD2; mature oligodendrocytes (MOL), PLP1 and MBP; oligodendrocyte precursor cells (OPCs), OLIG1 and OLIG2; astrocytes (Astro), GFAP and AQP4; microglia (Micro), CSF1R and CD74; endothelial cells (Endo), FLT1 and CLDN5; and pericytes (Peri), DCN and COL1A2. Cell clusters showing inconsistent expression of canonical lineage markers or poor transcriptomic quality were excluded before downstream analyses.

**5. Exploratory cell-type-specific differential-expression analysis**

Differential-expression analysis between MDD and HC samples was performed separately within each major cell type using MAST. The original analysis was conducted at the individual cell/nucleus level using the following model:

zlm(~ condition + nCount_RNA + percent.mt + Sex, sca, method = "glmer", ebayes = TRUE)

where *condition* represented MDD versus HC, *nCount_RNA* represented the total UMI count per cell, *percent.mt* represented the proportion of mitochondrial transcripts, and *Sex* represented biological sex.

The disease-associated effect was evaluated using a likelihood-ratio test, and P values were adjusted for multiple comparisons using the Benjamini-Hochberg procedure. Genes satisfying FDR < 0.05 and |log2 fold change| > 0.25 were retained in the original exploratory screening analysis.

Importantly, donor identity was not explicitly incorporated into this model. Because multiple cells or nuclei originated from the same biological subjects, the assumption of independence among individual observations is not fully satisfied. Consequently, within-donor correlation may result in underestimated variance and inflated statistical significance. The resulting differentially expressed genes should therefore be interpreted as exploratory cell-level signals rather than as confirmatory donor-level disease associations.

For subsequent cross-dataset analyses, genes showing opposite directions of change between the MDD cell-level analysis and the TMS-stimulated rat cortical transcriptomic dataset were retained as exploratory candidate directional-overlap genes. These directional overlaps were used for hypothesis generation, overlap visualization, and functional enrichment analysis. They should not be interpreted as demonstrating molecular reversal by TMS in patients with MDD or TRD.

A donor-aware strategy, such as cell-type-specific pseudobulk differential expression or a mixed-effects model incorporating subject identity, would be required for confirmatory subject-level inference.

**6. Functional enrichment and pathway network analysis**

Functional enrichment analysis of candidate directional-overlap genes was performed using aPEAR v1.0.0 in R. Gene Ontology (GO) Biological Process terms enriched among the candidate genes were identified and subsequently organized according to their functional similarity.

aPEAR was used to reduce the redundancy inherent in conventional GO enrichment results by constructing similarity networks among significantly enriched terms and grouping highly related biological processes into functional clusters. Representative terms were selected to summarize each functional module. Enrichment results were visualized as pathway networks, in which related GO terms were connected according to their functional similarity. This analysis was performed separately for candidate directional-overlap genes identified in different cell populations, particularly excitatory and inhibitory neurons, to characterize the cell-type-specific biological processes represented in these exploratory gene sets.

**7. Exploratory single-cell metabolic flux analysis**

Cell-type-specific metabolic alterations associated with MDD were inferred from the single-cell transcriptomic data using single-cell Flux Estimation Analysis (scFEA). The metabolic network containing 168 metabolic modules and 22 super-modules was obtained from the scFEA reference model. Before scFEA analysis, cells with fewer than 500 detected genes were excluded. The normalized cell-by-gene expression matrix was extracted from the Seurat object and used as input for the Python implementation of scFEA.

Metabolic fluxes were estimated independently for individual cells according to the expression of genes associated with each metabolic module. The resulting metabolic flux matrix was imported into the single-cell metadata and matched to individual cells using cell barcodes.

In the original exploratory analysis, metabolic features were compared between MDD and HC groups within each annotated cell type using cell-level observations and the Wilcoxon rank-sum test implemented through the compare_means function. Donor identity was not explicitly included in these comparisons. Therefore, cells derived from the same donor were not statistically modeled as correlated observations.

Because the independence assumption of the cell-level Wilcoxon comparison is not fully satisfied in a multi-donor single-cell dataset, the resulting P values may be affected by pseudoreplication and should not be interpreted as confirmatory subject-level statistical evidence. Accordingly, the metabolic differences shown in Figure 4 are treated as exploratory, transcriptome-derived metabolic signals intended to identify potentially relevant metabolic processes rather than validated MDD-associated metabolomic alterations.

The analysis was used primarily to visualize cell-type-specific patterns potentially involving amino acid, lipid, neurotransmitter, and energy metabolism. Future confirmatory analyses should summarize or model the inferred metabolic features at the biological-subject level and explicitly account for donor dependence.

**8. Differential expression analysis of the TMS-stimulated rat cortical RNA-seq dataset**

Bulk RNA-sequencing data from the cortex of TMS-stimulated rats and corresponding control animals were obtained from GEO under accession number GSE230149. Differential gene expression analysis between TMS-stimulated and control groups was performed using DESeq2 v1.46.0. Raw gene-level count data were used as input to DESeq2. Library-size normalization was performed internally using the median-of-ratios method implemented in DESeq2. Gene-wise dispersion estimates were obtained and fitted using the negative binomial generalized linear model. Differential expression between the TMS and control groups was assessed using the Wald test, and P values were corrected for multiple comparisons using the Benjamini-Hochberg procedure. Genes meeting the predefined criteria of FDR < 0.05 and |log2 fold change| > 1 were considered significantly differentially expressed. Because the MDD single-cell datasets were derived from humans whereas the TMS RNA-seq dataset was generated from rats, rat genes were converted to their corresponding human orthologs before cross-species comparison. Only genes with identifiable orthologous counterparts were retained for subsequent analyses. TMS-induced genes were then intersected with cell-type-specific MDD DEGs. Genes downregulated in the exploratory MDD analysis but upregulated in the TMS dataset, or genes upregulated in the exploratory MDD analysis but downregulated in the TMS dataset, were retained as candidate directional-overlap genes.

**9. Integration with published spatial transcriptomic data**

Spatial transcriptomic results describing cortical transcriptional responses to TMS were obtained directly from the corresponding published study. No reprocessing of the original spatial transcriptomic sequencing data was performed in the present study. Differentially expressed genes reported for individual cortical layers/regions and TMS stimulation paradigms were extracted from the published results and used for integrative comparison with the MDD single-cell transcriptomic data. Detailed procedures for spatial transcriptomic tissue processing, sequencing, quality control, spatial domain/layer identification, normalization, and differential expression analysis are therefore available in the original publication and its supplementary materials. For the present integrative analysis, published TMS-responsive genes from individual cortical spatial domains were compared with cell-type/subtype-specific MDD DEGs. Genes showing opposite directions of alteration between MDD and TMS stimulation were retained to construct the spatial domain-gene-cell subtype relationships shown in Figure 2.

**10. Integration with published proteomic datasets**

Published proteomic findings from postmortem prefrontal and anterior cingulate cortex samples of patients with MDD were extracted from the studies cited in the main manuscript. Protein-level differential-expression results were retained using the significance criteria reported in the original studies. Protein identifiers were harmonized to gene symbols where possible to permit comparison with the TMS-stimulated rat cortical RNA-seq dataset after rat-to-human ortholog mapping. Direction-aware comparisons were then performed between MDD-associated protein changes and TMS-related transcriptomic changes. Molecules showing opposite directions of alteration across the MDD proteomic and TMS transcriptomic datasets were retained as candidate TMS-related molecules and used to generate Figure 3. Because the comparison integrates different molecular layers, species, and experimental contexts, these overlaps were interpreted as exploratory and hypothesis-generating rather than as evidence of protein-level reversal by TMS in patients.

**11. Interpretation of metabolite-related analyses**

Figure 4 was derived from scFEA-based inference of cell type-specific metabolic fluxes from the integrated MDD single-cell transcriptomic datasets, as described in Section 7, rather than from direct metabolomic measurements in the same samples. Metabolite and pathway-level findings from published in vitro, animal, and peripheral clinical studies were used only for contextual comparison in the narrative review and were not pooled quantitatively with the scFEA results. Accordingly, the metabolic findings presented in Figure 4 should be interpreted as transcriptome-based metabolic inference and hypothesis-generating evidence.
